# Vegetation, topography, and soil depth drive microbial community structure in two Swedish grasslands

**DOI:** 10.1093/femsec/fiad080

**Published:** 2023-07-20

**Authors:** Daniela Guasconi, Jaanis Juhanson, Karina E Clemmensen, Sara A O Cousins, Gustaf Hugelius, Stefano Manzoni, Nina Roth, Petra Fransson

**Affiliations:** Department of Physical Geography and Bolin Centre for Climate Research, Stockholm University, 106 91 Stockholm, Sweden; Department of Forest Mycology and Plant Pathology, Uppsala BioCenter, Swedish University of Agricultural Sciences, 750 07 Uppsala, Sweden; Department of Forest Mycology and Plant Pathology, Uppsala BioCenter, Swedish University of Agricultural Sciences, 750 07 Uppsala, Sweden; Department of Physical Geography and Bolin Centre for Climate Research, Stockholm University, 106 91 Stockholm, Sweden; Department of Physical Geography and Bolin Centre for Climate Research, Stockholm University, 106 91 Stockholm, Sweden; Department of Physical Geography and Bolin Centre for Climate Research, Stockholm University, 106 91 Stockholm, Sweden; Department of Physical Geography and Bolin Centre for Climate Research, Stockholm University, 106 91 Stockholm, Sweden; Department of Forest Mycology and Plant Pathology, Uppsala BioCenter, Swedish University of Agricultural Sciences, 750 07 Uppsala, Sweden

**Keywords:** 16S, grassland, ITS, mycorrhizal fungi, plant community, saprotrophic fungi

## Abstract

Soil microbial diversity and community composition are shaped by various factors linked to land management, topographic position, and vegetation. To study the effects of these drivers, we characterized fungal and bacterial communities from bulk soil at four soil depths ranging from the surface to below the rooting zone of two Swedish grasslands with differing land-use histories, each including both an upper and a lower catenary position. We hypothesized that differences in plant species richness and plant functional group composition between the four study sites would drive the variation in soil microbial community composition and correlate with microbial diversity, and that microbial biomass and diversity would decrease with soil depth following a decline in resource availability. While vegetation was identified as the main driver of microbial community composition, the explained variation was significantly higher for bacteria than for fungi, and the communities differed more between grasslands than between catenary positions. Microbial biomass derived from DNA abundance decreased with depth, but diversity remained relatively stable, indicating diverse microbial communities even below the rooting zone. Finally, plant-microbial diversity correlations were significant only for specific plant and fungal functional groups, emphasizing the importance of functional interactions over general species richness.

## Teaser

Fungal and bacterial community composition across a soil depth profile in two Swedish grasslands is driven by vegetation and topography, but the drivers of abundance and diversity differ between groups.

## Introduction

Soil microbial communities play an important role in grassland ecosystem functioning, from nutrient cycling to carbon (C) sequestration (Prommer et al. 2020). By breaking down and converting organic matter into plant-available nutrients and by establishing symbioses with plant roots, they can enhance soil fertility and contribute to ecosystem health (Morris et al. 2020). However, these processes can be affected by biodiversity loss and by shifts in microbial community composition driven by land use change (Sala et al. 2000, Cao et al. 2017). While human land use affects ca. 70% of Earth’s surface and exerts pressure on global ecosystems, its effects on grasslands are particularly strong. Grasslands cover ca. 40% of the ice-free terrestrial surface (Sala et al. 2017), and grazing and mowing are among the most spatially extensive land use practices (Erb et al. 2017). Conversions from grassland to cropland, fertilization or intensive grazing can have long-lasting consequences for microbial communities and on the soil processes they mediate (Buckley and Schmidt 2001, Steenwerth et al. 2002, Habekost et al. 2008). Sweden has a long tradition of grassland management (Eriksson and Cousins 2014). Historically, grasslands have been used to support livestock for dairy and meat products, and the manure from livestock to fertilize crop fields. However, over the past century, the abandonment of traditional grassland management practices, coupled with the conversion of vast areas to croplands and forests, has significantly reduced the extent of present-day grasslands in Sweden to a fraction of their original size (Eriksson and Cousins 2014). Additionally, former crop fields are sometimes repurposed and managed as grasslands, resulting in the emergence of a new type of grassland. These cropland-to-grassland conversions could lead to increased organic matter inputs and plant–soil interactions (Yang et al. 2021). While grassland plant communities have been extensively studied, less is known about the status of microbial communities in Swedish grasslands on former arable fields and how they relate to existing plant communities.

Interactions between soil microorganisms and plant communities through positive or negative feedback depend on plant species composition and diversity (Ettema and Wardle 2002, van der Heijden et al. 2008, Bever et al. 2013). While general correlations between aboveground and belowground species diversity may not be evident (Hooper et al. 2000), certain functional groups of fungi and bacteria, such as pathogens and root-associated organisms like arbuscular mycorrhizal fungi, have shown a positive association with grassland plant biomass and species richness (Wardle et al. 2004, Van Der Heijden et al. 2006, Bever et al. 2013, Hiiesalu et al. 2014). This suggests that the nature and strength of the correlation between plant species richness and microbial diversity might differ between microbial functional groups. Furthermore, bacterial and fungal communities in soils can be expected to exhibit high variation across scales (Nunan et al. 2020), across both natural and anthropogenic (land use) gradients (Ettema 2002). For example, topographically driven variability in soil conditions explains differences in microbial community composition expressed in relative abundance of specific fungal functional groups (Schlatter et al. 2018), and poorly drained lower catenary positions can have higher microbial biomass compared to summits and slopes (Watts et al. 2010). Microbial composition differences may then be explained by higher soil moisture and increased availability of C and nutrients due to both transport processes and local retention mechanisms at lower catenary positions. Microbial communities also vary vertically in the soil profile. Microbial abundance is generally higher in the upper topsoil layers where root densities are highest (Berg et al. 1998, Xu et al. 2013) and in the rhizosphere (Lv et al. 2023) compared to bulk soil and decreases with soil depth as root biomass declines (Fierer et al. 2003, Xu et al. 2013). The resulting vertical stratification of microbial communities is determined by differences in substrate availability, soil horizon properties, and soil aggregate sizes (Fox et al. 2018). This variability is largely dependent on plant species identity and root distribution and activity, since plants provide soil microbial communities with substrates through aboveground and belowground litter input and root exudation (Wardle et al. 2004, Zhao et al. 2021). As different microbial functional groups vary in their response to substrate availability, the decline in root biomass and in available C and nitrogen (N) with depth leads to shifts in microbial community composition, and may, for example, result in decreasing fungal diversity (Schlatter et al. 2018). However, only a few studies have investigated how soil fungal and bacterial communities change with soil depth below the rooting zone in grasslands, although communities at different depths may play distinct roles for C and nutrient cycling.

Land management, plant community composition, topography, and soil depth may all be important in shaping microbial diversity and function. Here, we characterized the microbial community composition of two grasslands with different land-use histories, which reflected differences in the plant species richness, plant and functional group composition, and proximity to the forest border. By selecting two colocated grasslands with the same climate and similar in soil characteristics, such as pH, soil texture, and nutrient availability, we were able to control for potentially confounding effects. In each grassland, the fungal and bacterial communities were obtained from bulk soil at an upper and a lower catenary position, i.e. sites with similar soil parent material but different slope positions. Soils were sampled at four depths ranging from 0 to 50 cm, with the deepest soil layer containing <5% of the total root biomass. We investigated fungal and bacterial community composition in relation to edaphic properties and vegetation to identify potential drivers of fungal and bacterial diversity in grasslands and to test hypotheses that: (i) vegetation, catenary position, and soil depth affect the variation in soil fungal and bacterial community composition; (ii) with reference to soil depth in particular, microbial biomass abundance and diversity decrease with soil depth following a decline in resource availability; and (iii) microbial community diversity correlates with plant diversity and plant functional group composition.

## Methods

### Site description and sampling

The sampling plots were established in 2019 in two grasslands in proximity to Tovetorp Research Station (south-central Sweden, Table S1). The two grasslands (Tovetorp and Ämtvik) are located <4 km apart with similar soil parent material (post-glacial silty clay) and a humid continental climate with a mean annual precipitation of 619 mm and a mean annual temperature of 7.3°C (http://weather.zoologi.su.se). Both grasslands are former plowed arable fields, but with differences in time and management since cessation of agriculture. The Tovetorp location has been a conventionally plowed agricultural field until 1993, and later occasionally mowed and grazed by fallow deer. The Ämtvik location used to be a crop field belonging to a small farm, but was converted into a grassland and extensively grazed by cattle or mowed for at least 40 years prior to the study (information retrieved from local farmers). While the aboveground biomass does not differ significantly between the two grasslands, Tovetorp has a higher mean root biomass (Table S6). The vegetation in both grasslands is dominated by perennial grasses, but Ämtvik hosts considerably more forbs and Leguminosae (26% of the total aboveground plant biomass) than Tovetorp (9%), and has a higher mean plant species richness (Table S6). The sampling sites in Ämtvik are also closer to the edge of a mixed deciduous and coniferous forest (6–20 m, compared to 50–90 m in Tovetorp).

In each grassland, 12 plots (2 × 2 m) were placed 0.5–2 m apart at a higher catenary position, and 12 similar plots were placed at a lower catenary position (hereafter called high/low elevation sites; see Fig. S1). While these paired high/low elevation sites were only ∼50 m apart and elevation differed only by ∼6 m, the lower elevation sites had on average ca. 10% higher volumetric soil moisture measured at 60 cm depth. Vegetation inventories were carried out throughout the growing season of 2019 (April to October) to capture the maximum coverage of each species. The presence of all plant species was recorded in each plot, and their coverage was estimated to the nearest 1%. In addition to plant species richness and diversity calculated as Shannon–Wiener diversity index (*H*’), aboveground biomass (reported as dry weight) was measured in mid-July 2019 by harvesting all the vegetation from one quarter (1 m^2^) of every plot by cutting at ground level, including moss and dead plant biomass. In half of all plots, the biomass from a 50 × 50 cm square was sorted into grasses, forbs, and legumes to obtain their relative abundance in dry biomass (moss, woody plants, and dead plant biomass were measured, but excluded from the analyses). Distance from the closest trees (m) was measured to the center of each plot. Root biomass in the upper 30 cm was obtained from one soil core (8 cm diameter) sampled from each plot in September 2019, and the dry weight was recorded after rinsing the roots from soil by rinsing them with water on a 0.5 mm mesh sieve, followed by drying at 60°C for at least 48 h.

Soil moisture was measured every 3 weeks throughout the growing season from one access tube (1 m long) permanently installed in each plot, using a PR2 profile probe (Delta-T Devices Ltd, Cambridge, UK). The values used in the analyses are growing season averages of volumetric soil water content (%) in each plot, measured every 10 cm in the first 50 cm. One soil sample for elemental and DNA-based analyses was collected in each plot in July and August 2019 with a Pürckhauer soil corer (2.5 cm diameter; Eijkelkamp, The Netherlands) from four depths (0–10 cm, 10–20 cm, 20–30 cm, and 40–50 cm) and immediately stored frozen. A portion of each sample was used for pH measurements (Mantech Automax 73, Guelph, ON, Canada), while the rest was freeze-dried, ground, and sieved. A subsample was sent to the Stable Isotope Facility at UC Davis (California) for estimation of total C and N contents (µg/mg dry weight soil). Soil organic matter (SOM; %) was estimated from total organic matter content after loss on ignition (550°C) from three additional soil samples collected from each site in September. The samples for the DNA analyses were obtained by grouping the 12 plots of each grassland and elevation into three blocks and pooling the soil samples of the four adjacent plots of each block for each depth, thus resulting in a total of 48 pooled samples (three replicate samples per grassland, elevation, and depth).

### DNA extraction and quantification of fungi and bacteria

DNA was extracted from a subsample of ∼100 mg of soil for soil depths 0–10 cm, 10–20 cm, and 20–30 cm and 400 mg for the deepest soil samples (40–50 cm) using the Macherey–Nagel NucleoSpin Soil Kit (Düren, Germany), with the following specifications: For all samples from 0 to 30 cm depth, 700 µl lysis buffer SL1 and 150 µl enhancer SX were used, and for the deep soil samples, reagent quantities were adjusted for the larger soil weight to 1000 µl of lysis buffer SL1 and 214 µl of enhancer SX. First, the soil samples went through mechanical lysis with ceramic beads in a bead beater (Precellys, Bertin Technologies SAS, FR), and the extracted DNA was quantified using a NanoDrop spectrophotometer (Thermo Scientific, Wilmington, DE, USA) and diluted to 1 ng/ul for fungal and bacterial PCRs.

The abundances of fungal and bacterial communities were estimated by quantifying the ITS2 region and the 16S rRNA gene, respectively. Quantitative PCR analyses were run in duplicates in 20 µl reactions using 2 ng DNA template, IQ SYBR green supermix, and bovine serum albumin (BSA; 0.1%), as described in Castaño et al. (2022). Primers used for fungal qPCR were forward primer gITS7 (GTGARTCATCGARTCTTTG; Ihrmark et al. 2012) and reverse primers ITS4 (75%; 5′-TCCTCCGCTTATTGATATGC-3′, White et al. 1990) and ITS4a (25%; 5′-CACACGCTGTCCTCGCCTTATTGATATGC-3′, Sterkenburg et al. 2015), and primers for bacterial qPCR were forward primer Pro341F (CCTACGGGNBGCASCAG; Takahashi et al. 2014) and reverse primer 534R (ATTACCGCGGCTGCTGG; Muyzer et al 1993). Before the analyses, PCR inhibition tests were run by adding a known quantity of standard plasmid (pGEM-T plasmid), which showed no significant inhibition by sample extracts. The reactions were run on a quantitative PCR cycler (BioRad iQ5, Life Technologies, Carlsbad, CA, USA), with cycling conditions of 5 min at 95°C, 39 cycles of [15 s at 95°C, 30 s at 56°C, 40 s at 72°C, and 5 s at 78°C]. Standard curves for quantifications were obtained by serial dilutions of linearized plasmids containing either the ITS2 or 16S marker. The resulting copy numbers were corrected for the amount and the concentration of the DNA extracted from the original soil samples and calculated per g of dry soil. The fungal: bacterial ratio was calculated based on ITS and 16S rRNA gene copy numbers obtained through qPCR.

### Sequencing of fungal and bacterial communities

Fungal and bacterial libraries were prepared by amplifying the ITS region and 16S rRNA gene, respectively, by using the same primers as for the qPCR for fungal ITS2 amplicons (see above), and forward primer Pro341F (CCTACGGGNBGCASCAG) and reverse primer Pro805R (GACTACNVGGGTATCTAATCC; Takahashi et al. 2014) for the bacterial V3–V4 region amplicons. The ITS2 primers contained unique identification tags for each sample (Clemmensen et al. 2016), while the 16S rRNA-specific primers contained Nextera adaptor sequences (Illumina, San Diego, CA, USA). PCR for fungal DNA amplicons was performed in duplicates of 50 µl reactions with 25 ng of DNA template, 0.2 mM of each nucleotide, 2.75 mM MgCl_2_, 0.5 µM of gITS7 primer, 0.3 µM of ITS4 primer, 0.1 µM of ITS4a primer, and 0.025 U µl^−1^ DreamTaq polymerase in 1 X DreamTaq buffer (Thermo Scientific, MA, USA). PCR conditions were 5 min at 95°C, 25–35 cycles of [30 s at 95°C, 30 s at 56°C, and 30 s at 72°C] and 7 min at 72°C. The PCR products were purified using the AMPure kit (Beckman Coulter, CA, USA). The bacterial libraries were prepared using a two-step PCR protocol. The first PCR used a 15 µl reaction in duplicates with 4 ng of DNA template, BSA (20 µg µl^−1^), and 0.25 µM of each primer in 1 X Phusion High Fidelity PCR Master Mix (Thermo Scientific, MA, USA), followed by a second (indexing) PCR using 3 µl of purified product from the first PCR as template, BSA, polymerase, and 0.2 µM of each forward and reverse multiplexing primers (containing Nextera barcoding regions for dual labeling). PCR conditions for the first reaction were 3 min at 98°C, 25 cycles of [15 s at 98°C, 30 s at 55°C, and 40 s at 72°C] and 10 min at 72°C, for the second (in which sample tags are added) 3 min at 98°C, 8 cycles of [30 s at 98°C, 30 s at 55°C, and 45 s at 72°C] and 5 min at 72°C. All PCR runs were run on a SimpliAmp™ Thermal Cycler (Thermo Scientific, MA, USA). Bacterial PCR products were purified using Sera-Mag™ magnetic beads (GE Healthcare, IL, USA).

The final PCR products for fungi and bacteria were quantified using a Qubit® 2.0 fluorometer (Invitrogen, Carlsbad, CA, USA), pooled in equal DNA quantities, and the resulting pools cleaned using the E.Z.N.A.® Cycle Pure Kit (Omega Bio-Tek, Norcross, GA, USA). For the fungal pool, adaptor ligation and sequencing were performed by NGI-Uppsala/SciLifeLab (National Genomics Infrastructure, Uppsala, Sweden) on a PacBio Sequel instrument (Pacific Biosciences, Menlo Park, CA, USA) using one Sequel SMRT cell (v3). The bacterial pool was sequenced on the MiSeq platform using the 2 × 250 bp paired-end chemistry (Illumina, San Diego, CA, USA).

### Sequence processing and taxonomic classification

Fungal raw sequence counts were analyzed using the bioinformatics pipeline SCATA (https://scata.mykopat.slu.se), where sequences were quality filtered, screened for primers and identification tags, and clustered according to Kyaschenko et al. (2017). Sequences with mean quality of <20 or containing single bases with quality of three or less were removed, as well as all sequences containing mismatched identification tags. Sequences were clustered into study-level species hypotheses (SHs; Kõljalg et al. 2013) based on single-linkage clustering with a 1.5% threshold dissimilarity for sequences to enter an operational taxonomic unit (OTU). Fungal and nonfungal sequences were identified using BLASTn using the PlutoF platform (Abarenkov et al. 2010) in the UNITE database (fungal database, version 8.4), which bases identifications on SHs, and by constructing neighbor-joining trees in the MEGAN community edition (version 6.20.2019; Huson et al. 2011, Bálint et al. 2014). Representative sequences for the fungal OTUs were assigned to appropriate taxonomic levels depending on the similarity of the representative sequence (>97% match for species-level identification, 90% for genus, 85% for family, 80% for order, 75% for class, and 70% for division/phylum).

For fungi, sequencing of the dataset generated a total of 252 114 sequences, of which 163 904 passed quality control (QC) and were clustered into a total of 1619 OTUs. A total of five hundred fifty-two nonfungal OTUs (22 264 sequences, 24% of counts that passed QC) were removed, as well as all OTUs in the global dataset with three counts or less, and 11 OTUs present only in the negative controls with small count numbers. The final fungal dataset consisted of 780 OTUs across all samples (69 292 sequence counts), for which relative abundances were calculated per sample. To account for differences in fungal abundance and thus in the amount of DNA extracted from each soil sample in the analyses, and as a better representation of fungal biomass, the relative species abundances were also multiplied by the total number of fungal copies per sample resulting from the qPCR (after eliminating the nonfungal proportions), as described in Parker et al. (2022). Identification of fungal functional groups was done using the FungalTraits database (Põlme et al. 2020) followed by careful manual curation, where unmatched sequences were classified into functional guilds based on taxonomic identity or similarity to database sequences recorded from well-defined substrates, if possible. About half of the OTUs (395 OTU) corresponding to almost 95% of the total sequences (65 769 sequences) were assigned to 12 functional groups based on primary lifestyle (animal parasites, mycoparasites, plant pathogens, endophytes, lichenized and lichen parasites, wood saprotrophs, litter saprotrophs, dung saprotrophs, soil saprotrophs, undefined saprotrophs, arbuscular mycorrhizae, and ectomycorrhizae), whereas the remaining 385 were labeled as “unknown” regarding their ecology.

The 16S rRNA raw reads were first trimmed using the FASTX-toolkit (http://hannonlab.cshl.edu/fastx_toolkit) and then merged using PEAR (Zhang et al. 2014). Removal of the chimeras was performed with UCHIME (Edgar et al. 2011), and dereplication and clustering of the sequences into OTU at 98% similarity threshold was performed with VSEARCH (Rognes et al. 2016). OTU clusters with <2 reads across all samples were discarded. Representative sequences for each OTU were aligned and classified with the SILVA Incremental Aligner (SINA) using the SILVA 138 database as a reference (Yilmaz et al. 2014). OTUs identified as mitochondria and chloroplasts as well as eukaryotes were discarded resulting in a final dataset consisted of 6 975 146 sequence counts (excluding mitochondria and chloroplasts) grouped clustered into 7115 OTUs. Species accumulation curves were plotted for all samples (Fig. S2).

Fungal and bacterial sequences are available in the NCBI Sequence Read Archive (www.ncbi.nlm.nih.gov/sra) under the accession number PRJNA938302.

### Statistical analyses

Differences in soil properties and vegetation and differences in microbial DNA copy numbers obtained through qPCR (hereafter called “abundance”) between depths, grasslands, and elevations were tested with three-way ANOVAs (the microbial abundance after being rank-transformed). The dissimilarities between microbial fungal and bacterial community composition between grasslands, elevations, and soil depths were tested in R (version 3.3.3; R core Team 2017) by Analysis of Similarity (ANOSIM; R package: vegan, Oksanen et al. 2020). To further test which fungal divisions, bacterial phyla, and fungal functional groups differed in abundance between grasslands, elevations, and soil depths, we used the *mvabund* function (R package: mvabund, Wang et al. 2021). This test is used to fit generalized linear models to each response variable (each species or group) and is suited for microbial species-abundance datasets.

The total fungal and bacterial community datasets were further analyzed using ordination methods in CANOCO 5.02 (Microcomputer Power, Ithaca, NY, USA). Graphical representation of the variation in community composition between grasslands, elevations, and soil depths was obtained with a principal correspondence analysis (PCA) summarizing the variation in species composition among samples. For the fungal communities, the PCA was based on the relative abundance of 780 identified fungal OTUs, and data were not rarefied. For the bacterial communities, the PCA was based on the relative abundances of 7115 identified OTUs, and data were rarefied by scaling with ranked subsampling according to Beule and Karlovsky (2020) to 59 701 sequences per sample. Correlation of fungal or bacterial communities with edaphic and plant variables was evaluated for significance using redundancy analysis (RDA) with forward selection and Monte Carlo permutations (without permutations within plots to account for dependency between soil depths from the same plot), in order to test potential drivers of the microbial communities. RDA analyses testing only for effects of the categories grassland, elevation, and soil depth (sample categories), preceded testing the edaphic and plant variables (continuous variables). Vectors in the PCA species plots (unconstrained analysis with supplementary variables function in CANOCO) indicate direction and degree of correlation between the PCA axes and the significant edaphic and plant variables, which were selected based on significance in the RDA analyses. One sample was lost during fungal sequence processing, resulting in two replicates instead of three for the treatment Tovetorp low elevation (at 10–20 cm depth) and 47 fungal communities in total. The missing sample was replaced in the fungal RDA analysis by the mean number of counts and corresponding mean values for plant and soil variables based on the two replicates within the same treatment, in order to achieve a balanced split-plot design to account for soil depth dependency. The fungal and bacterial communities at the four soil depths were further tested separately with individual RDA analyses to evaluate whether potential drivers of community composition depended on soil depth. For all analyses, sequencing output per sample was accounted for by including sequence counts as a covariate. Both fungal and bacterial communities were also analyzed using nonmetric multidimensional scaling (NMDS) summarizing the similarities in species composition between samples for comparison, since this method is commonly used for bacteria.

Fungal and bacterial OTU numbers (hereafter “species richness”) and Shannon–Wiener diversity index, which reflects both richness and species relative abundances (*H*’, hereafter “diversity”), were calculated for each sample for fungi (rarefied to 592 counts per sample) and bacteria (rarefied to 59 701 counts per sample), and for the major fungal functional groups (saprotrophs, pathogens, and mycorrhizae) and the most abundant fungal and bacterial phyla (Ascomycota and Basidiomycota for fungi, Actinobacteriota, Proteobacteriota, Verrucomicrobiota, Acidobacteriota, and Chloroflexi for bacteria). To test for differences in species richness and diversity between grasslands, elevations, and soil depths, we used *t*-tests or ANOVAs followed by Tukey’s HSD test. Correlations between microbial abundance and diversity with vegetation and edaphic variables were tested with a correlation matrix (Pearson correlation coefficient).

All predictive variables used in the statistical analyses were selected after checking for multicollinearity with a correlogram and by calculating the variable inflation factor, which excluded the following variables from further analyses: total plant cover, richness of grasses and forbs, pH, and soil C: N ratio. All analyses except for the ordination analyses (see above) were performed using R, and residuals were checked for normality.

## Results

### Site characteristics

**Figure 1.**
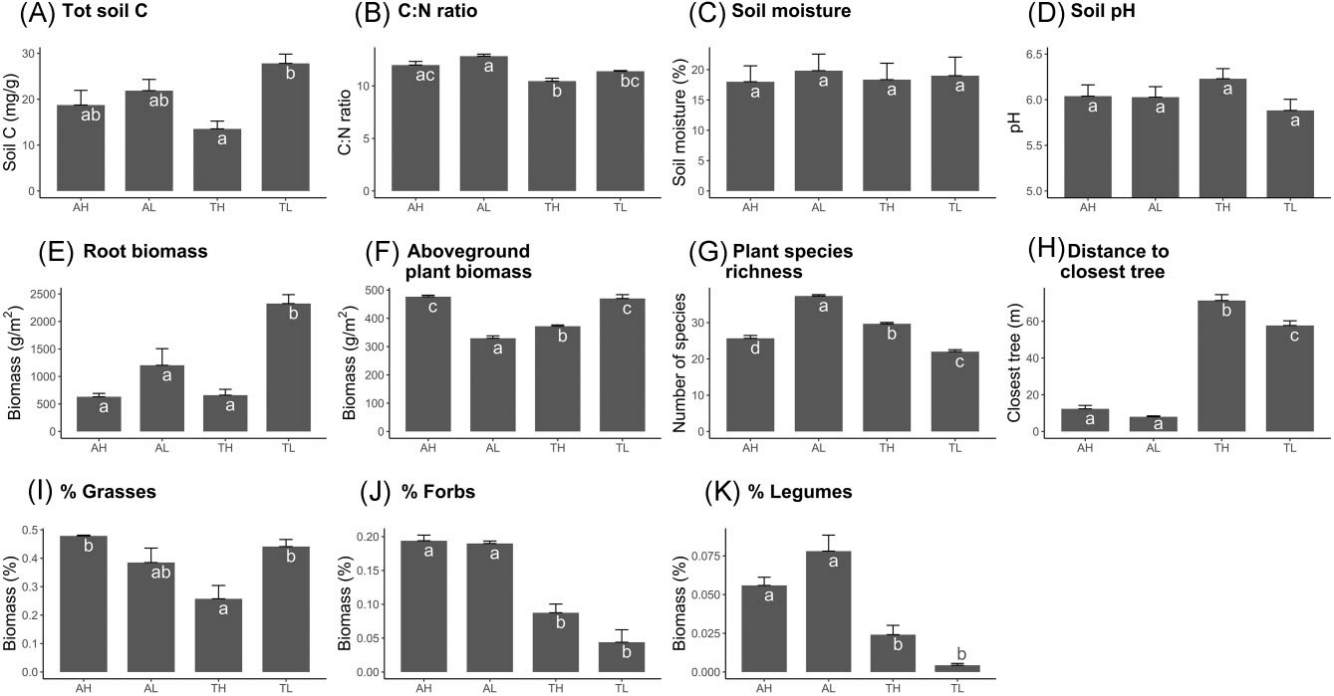
Soil and vegetation variables in two grasslands in south-eastern Sweden (A = Ämtvik and T = Tovetorp) at two catenary positions (H = high and L = low), given as mean values + SE: (A) total soil C, (B) soil C: N ratio (on a mass basis), (C) volumetric soil moisture (%), and (D) soil pH calculated from the mean values of the four depths considered (0–10 cm, 10–20 cm, 20–30 cm, and 40–50 cm) in each of the 48 plots (*n* = 48); (E) root biomass and (F) aboveground plant biomass; (G) plant species richness; and (H) distance to the closest tree in each plot (*n* = 12); percentage of aboveground biomass of (I) grasses, (J) forbs, and (K) legumes in half of the plots (*n* = 6) (percentages of moss, woody plants, and dead plant biomass not shown). Different letters indicate significant differences between groups (*P* < .05).

**Figure 2.**
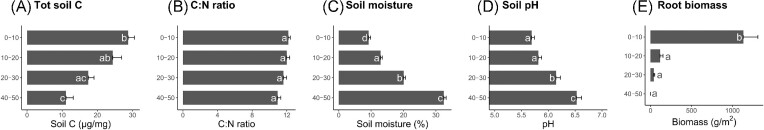
Soil and root variables in the soil layers 0–10 cm, 10–20 cm, 20–30 cm, and 40–50 cm, given as mean values (*n* = 48) + SE: (A) total soil C, (B) C: N ratio (on a mass basis), (C) volumetric soil moisture (%), (D) root biomass, and (E) soil pH. Different letters indicate significant differences between groups (*P* < .05).

The grasslands at lower elevation had higher soil C (*P* < .001) and root biomass (*P* < .001) compared to the grasslands at higher elevation (Fig. 1). Differences between Ämtvik and Tovetorp were mostly evident in the plant community composition (more forbs and legumes, *P* = .001 and *P* = .02, respectively) and stronger influence of nearby trees in Ämtvik. Soil C and root biomass decreased consistently with depth in the soil profile, whereas soil moisture and pH increased (*P* < .001 for all four variables, Fig. 2).

### Dissimilarity in microbial communities across sites, elevation, and soil depths

**Figure 3.**
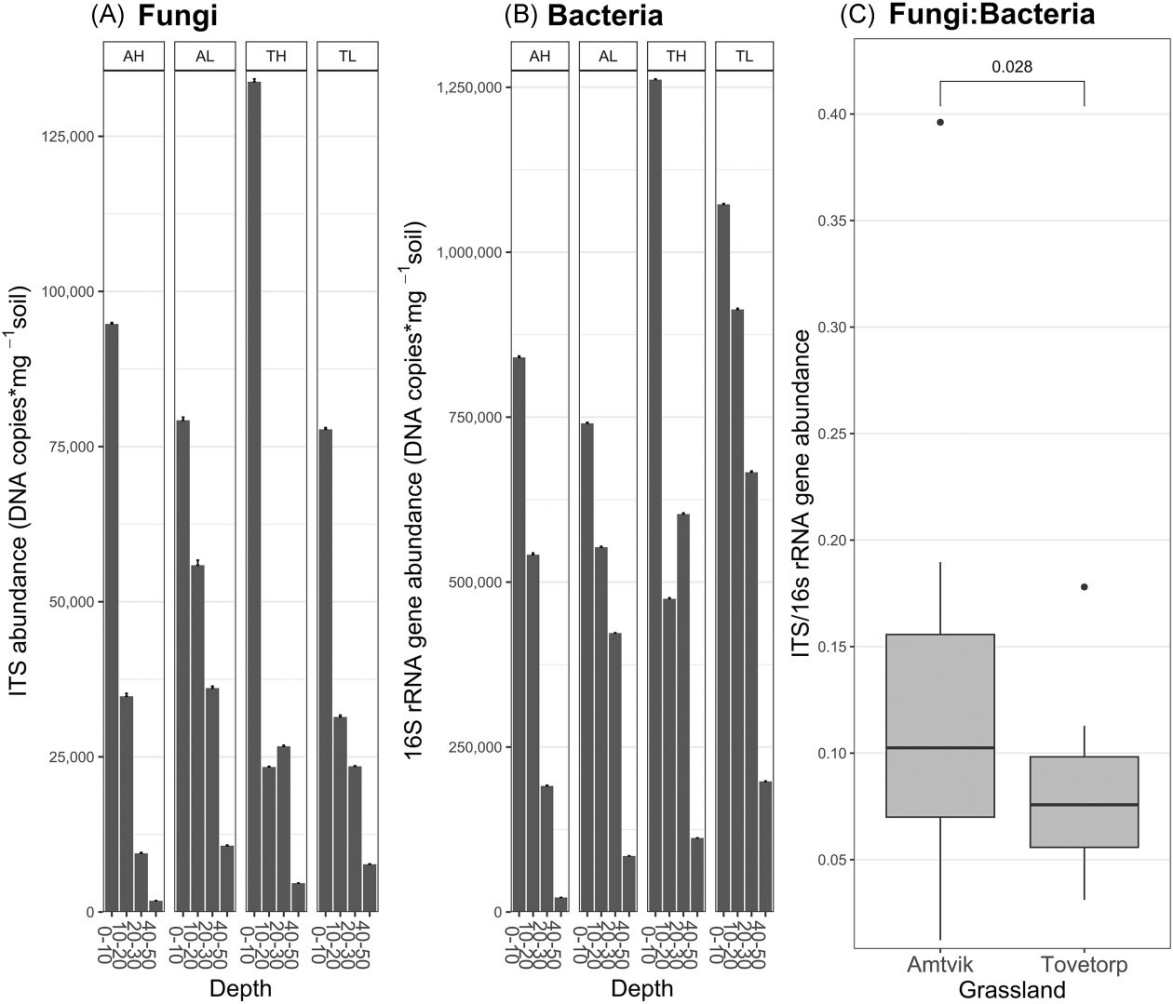
Abundance of total (A) fungi ITS region and (B) bacterial 16S rRNA gene at different soil depths and sites, in two grasslands in south-eastern Sweden (A = Ämtvik and T = Tovetorp) and two catenary positions (H = high and L = low); and (C) ratio of fungal to bacterial abundance in the Ämtvik (mean = 0.117) and Tovetorp grasslands (mean = 0.078; *t* = 2.3, *P* = .028). Fungal and bacterial abundances were obtained through qPCR (DNA copies*mg^−1^ of DNA markers in the soil) and given as mean values (*n* = 3) + SE.

The abundance of fungi and bacteria based on quantifications of marker genes decreased with soil depth (fungi: *P* < .001, bacteria: *P* < .001; Fig. 3A, B), but fungal abundance did not differ between grasslands and elevations. Bacterial abundance was higher in the Tovetorp grassland compared to Ämtvik (*P* < .01) and in the lower elevations (*P* = .04). Fungal and bacterial abundances were well correlated, so that the fungal: bacterial ratio did not differ between depths and elevations, even if it was overall higher in Ämtvik than in Tovetorp (*t* = 2.3, *P* = .028) (Fig. 3C).

**Figure 4.**
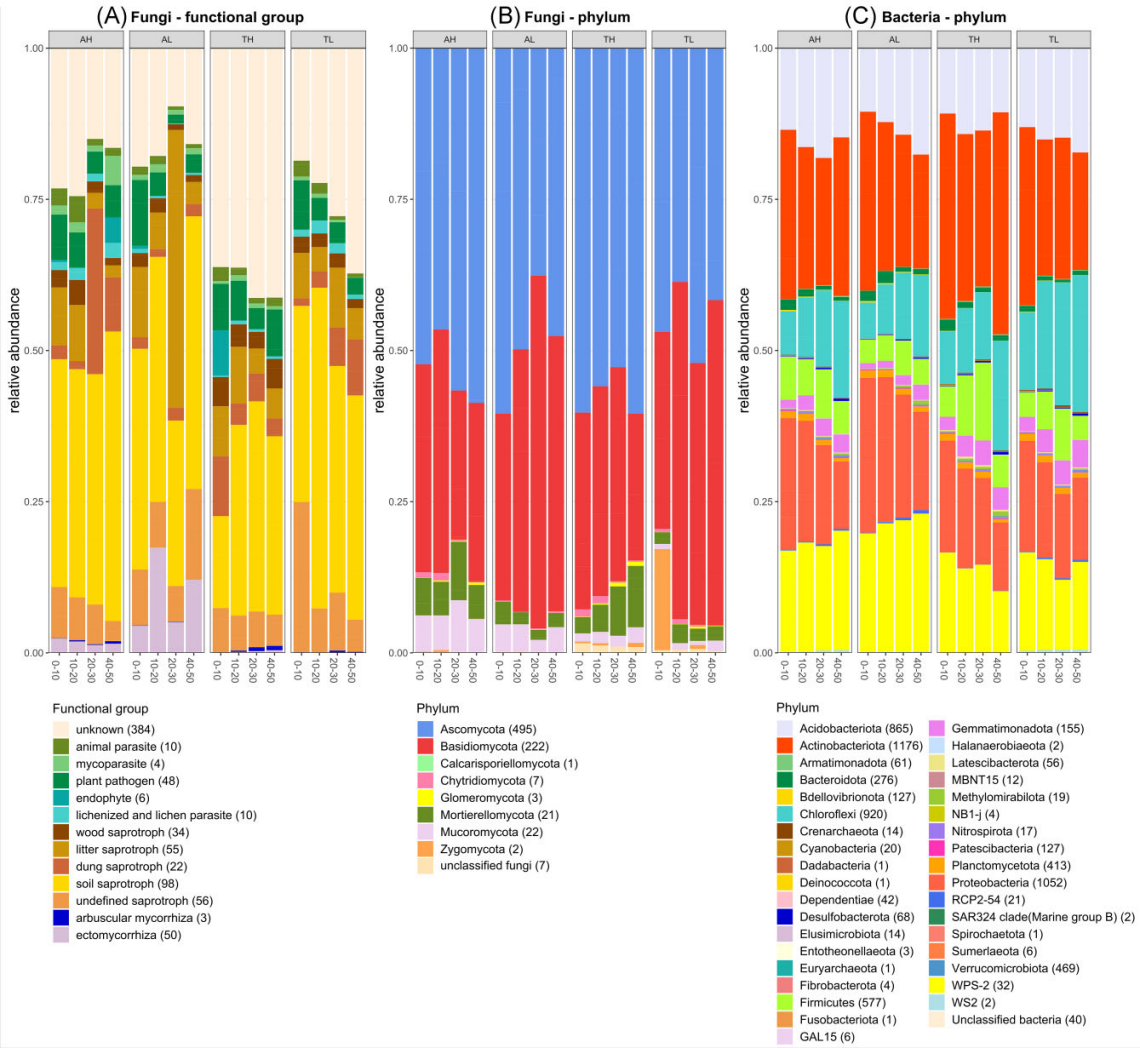
Relative abundances (*n* = 3) of fungal and bacterial taxonomic groups (phylum) and fungal functional groups over depths and sites, in two grasslands in south-eastern Sweden (A = Ämtvik and T = Tovetorp) and two catenary positions (H = high and L = low). Stacked bars represent the relative abundances of the different microbial taxonomic or functional groups as a fraction of total sequences grouped at functional or phylum level. Numbers in brackets indicate the number of OTUs assigned to each group. *P*-values from the Mvabund analyses are reported in Table S4.

The general trend of decreasing microbial abundance with depth but no significant difference in abundance between grasslands and elevations was true for most fungal functional groups and fungal and bacterial phyla, with the exception of mycorrhizal fungi (mvabund analyses, relative abundances presented in Fig. 4). Arbuscular mycorrhiza and ectomycorrhiza did not differ significantly in abundance through the depths considered, and only ectomycorrhiza differed significantly between grasslands, being predominantly present in Ämtvik (details in Table S4).

**Figure 5.**
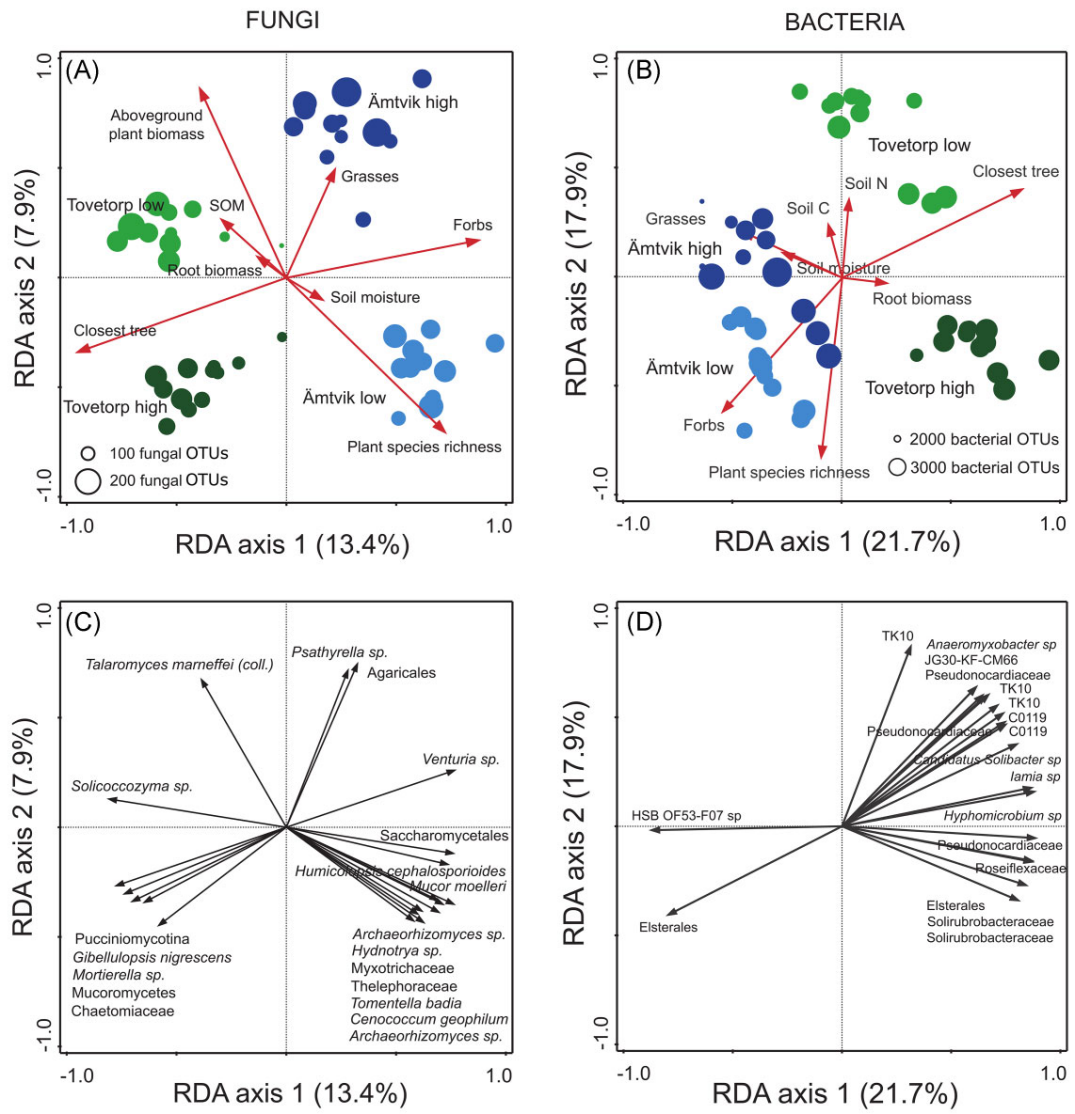
Variation in soil fungal and bacterial community composition and distribution of fungal (47 samples) and bacterial operation taxonomical units (OTUs; 48 samples) from the grasslands Tovetorp and Ämtvik in south-eastern Sweden visualized by (A, B) sample and (C, D) species plots of RDAs based either on PacBio sequencing of amplified ITS2 markers for fungi or Illumina MiSeq sequencing of amplified 16S rRNA gene markers for bacteria. The RDAs included 780 identified fungal OTUs (data not rarefied) and 7115 identified bacterial OTUs (data rarefied). Circles are color coded according to grassland and elevation. The size of circles in (A, B) corresponds to the number of OTUs in each sample. Red vectors in (A, B) represent constraining plant and soil variables, and indicate direction and degree of correlation between RDA axes and aboveground plant biomass, plant root biomass, number of plant species, proportion of grasses and forbs, distance to closest tree, soil moisture, and SOM content. Vectors in (D) represent constraining plant and soil variables, and indicate direction and degree of correlation between RDA axes and predictor variables. For the species plots (C, D) only the 30 most abundant OTUs are shown.

The Tovetorp and Ämtvik grasslands and the two catenary positions were colonized by distinct soil fungal (Figs 5A and S3a) and bacterial communities (Figs 5B and S3b). For fungi, the variation across depths and replicates was somewhat higher in Ämtvik, and elevation appeared to be a stronger driving force in Ämtvik compared to Tovetorp (Fig. 5A). For bacteria, community variation was higher at Tovetorp (Fig. 5B), and the high elevation emerged instead as clearly distinct from the other three communities. Grassland site explained 25.8% of the variation in fungal composition, while elevation explained 12.4% (RDA; categorical treatments, not shown). For bacterial communities, 43.6% and 20.4% of the variation were explained by grassland site and elevation, respectively (RDA; categorical treatments, not shown). Microbial communities also shifted with soil depth explaining an additional 3.9% of the variation for fungi (Fig. S3c) and an additional 10.7% of the variation for bacteria (Fig. S3d) (RDAs; grassland, elevation, and depth, not shown). Significant differences in community composition among grasslands, elevations, and depths were also identified in the NMDS (Fig. S4) and ANOSIM (Table S3) analyses, although the ANOSIM showed higher correlation coefficient values for depth compared to grassland and elevation. RDA analyses of bacterial communities generally explained twice as much of the variation in community composition than for fungi. When absolute community variation (i.e. relative abundances multiplied with total fungal copy numbers in samples derived by qPCR data, Figs S5 and S6) was tested in the same way, the resulting fungal compositional patterns stayed very similar, with the main difference being a clearer separation between Tovetorp high and low elevations. Variation in fungal and bacterial communities in the different grasslands and elevations was maintained at each individual soil depth (RDAs; Figs S7 and S8).

### Drivers of microbial community composition

Correlations between microbial community composition and edaphic and plant variables partly differed between fungi and bacteria. In the RDA analyses, the adjusted explained variation for plant and soil variables together was 26.5% for fungi and 49.4% for bacteria (Table S2). The drivers of fungal communities with the highest explanatory power in the RDA analyses with forward selection were distance to the closest trees (explaining 12.5% of the variation) and number of plant species (8.0%) (Fig. 5, Table S2). Fungal community variation also correlated significantly with plant root biomass, proportion of forb, and grass biomass, aboveground plant biomass, SOM, and soil moisture (growing season average). pH was significantly correlated with SOM, and did not improve the model when replacing SOM in the RDA. For bacterial communities, distance to closest trees (20.9%), number of plant species (15.0%), soil moisture (10.4%), and total soil N (8.5%) were the strongest drivers (Fig. 5, Table S2), and plant root biomass, proportion of forbs, and grasses biomass and soil C were also significant. The other drivers each explained 4.2% or less of the variation in microbial community compositions.

When each soil depth was tested independently, multivariate analyses revealed that distance to the closest tree and the number of plant species were significant drivers of both fungal and bacterial communities for all soil depths (Figs S7 and S8). For the bacterial communities, soil N was a significant driver for the two upper layers, and soil C for the two deeper soil layers.

### Drivers of microbial abundance, richness, and diversity

**Figure 6.**
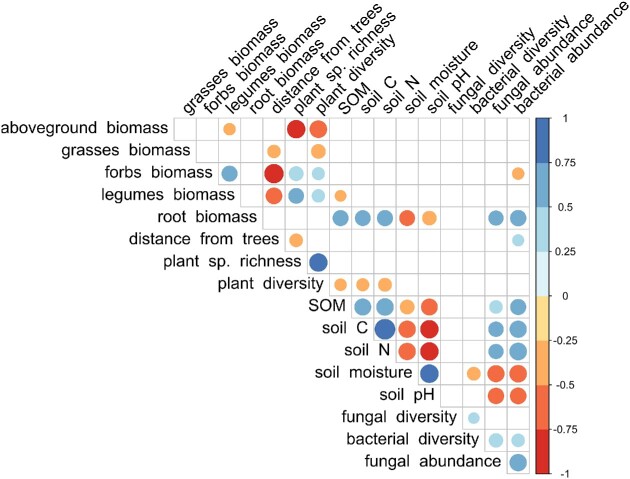
Correlation matrix displaying the relationship between the Shannon diversity index (*H*’) of fungi and bacteria, fungal and bacterial abundance (based on the qPCR analyses), and the edaphic and plant variables included in the study. The color scale indicates whether the correlation is positive (blue scale) or negative (red scale). Only statistically significant correlations are shown. The size of the dots indicates the *P*-value (reported in Table S5), with larger dots representing stronger significance. Fungal and bacterial diversity indexes are calculated on the rarefied datasets. Correlations with the diversity of broad taxonomic groups and fungal functional groups are reported in the Supplements.

**Figure 7.**
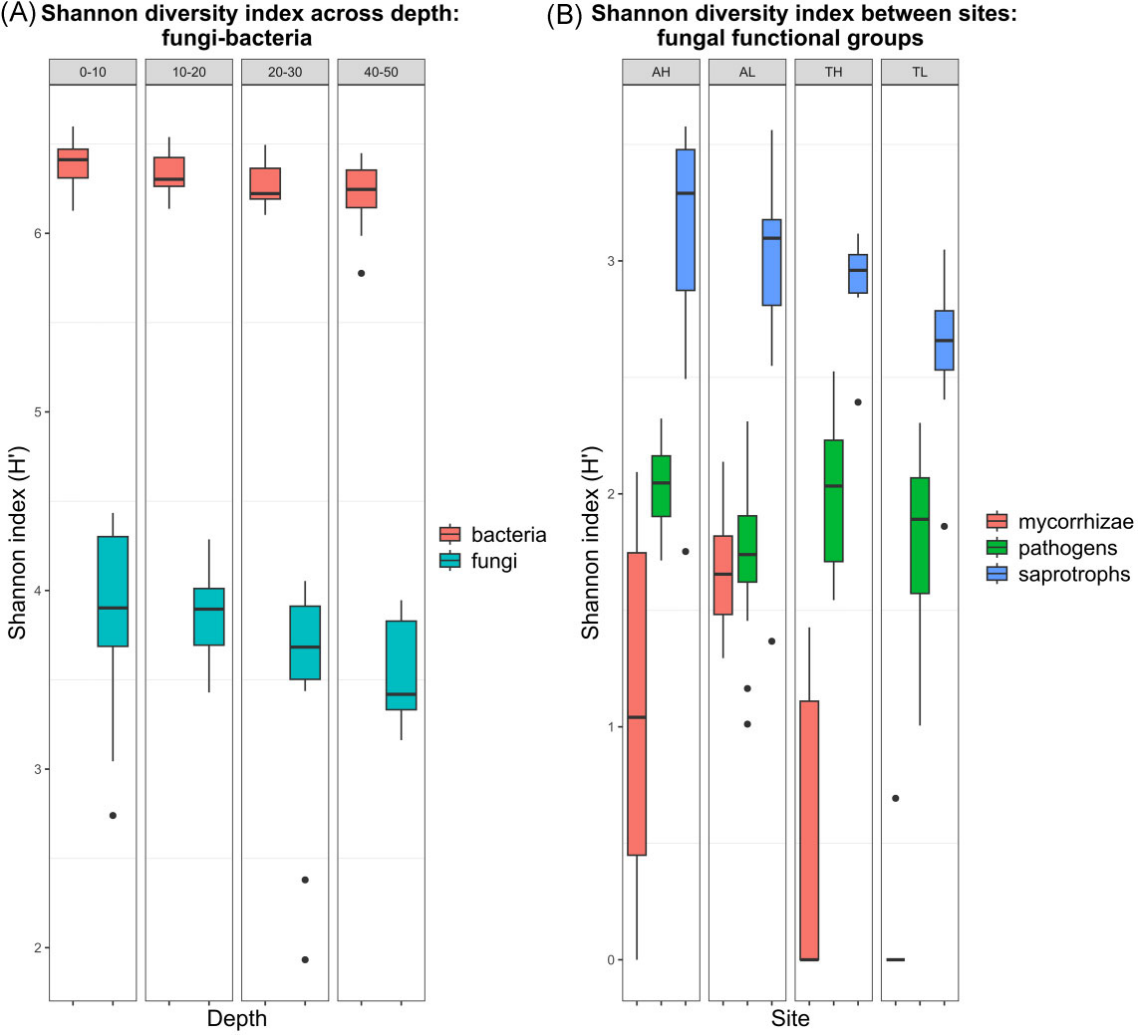
Shannon diversity index (*H*’) values for (A) fungi (mean *H*’ = 3.7) and bacteria (mean *H*’ = 6.3) at four soil depths and for (B) major fungal functional groups (mean *H*’ mycorrhizae = 0.8; pathogens = 1.9; and saprotrophs = 2.9) in two grasslands in south-eastern Sweden (A = Ämtvik and T = Tovetorp) and catenary positions (H = high and L = low). Bars show mean and interquartile range (IQR); whiskers extend to 1.5 x IQR; dots in the graph are outliers.

Bacterial and fungal abundances were positively correlated with root biomass and SOM content (and both C and N contents), but negatively correlated with soil moisture (Fig. 6), reflecting the vertical trends noted in Fig. 3. Bacterial communities had overall higher species richness than fungi (total mean sp. richness bacteria = 3145; total mean sp. richness fungi = 114) and higher diversity (*H*’ = 3.7 in fungi, *H*’ = 6.3 in bacteria; *P* < .001, Fig. 7A). Bacterial abundance and diversity decreased with depth (*P* = .01 and *P* = .037, respectively; the difference was significant only between the 0–10 cm and 40–50 cm soil depths, data not shown) and did not differ between grasslands. Further, bacterial diversity was negatively correlated with soil moisture (Fig. 6, Table S5a). Fungal diversity was similar across sites and soil depths (Fig. 7A) and was not significantly correlated with any variable, although fungal species richness declined somewhat with depth (*P* < .001, data not shown). Bacterial and fungal species richness, however, correlated to the same variables as bacterial and fungal abundances (positive correlations with SOM, total C, root biomass, and negative correlation with soil moisture, Table S5).

Diversity values differed also between the major fungal functional groups, in particular for mycorrhizal fungi compared to pathogens and saprotrophs. Mycorrhizal fungal diversity had the lowest values and differed significantly between the two grasslands (*P* < .001, mean *H*’ mycorrhizae in Ämtvik = 1.4 and Tovetorp = 0.3), while the diversity of pathogens and saprotrophs did not differ between the grasslands and elevations (Fig. 7B). None of the fungal groups differed significantly in diversity between depths. Further, the diversity of mycorrhizal fungi correlated positively with the proportion of forb and legume biomass, aboveground plant biomass, and distance from trees. In contrast, fungal saprotrophs correlated with forb biomass and were the only group that showed a positive correlation with plant diversity. Pathogen fungal diversity only correlated with soil moisture (Table S5b).

For fungi, diversity differed between the most abundant fungal divisions (mean *H*’ Ascomycota = 3.5, mean *H*’ Basidiomycota = 2.2; *P* < .001) and decreases with depth in Ascomycota (*P* < .001). None of the groups, however, differed between grasslands, and only Basidiomycota had higher diversity in the high-elevation sites (*P* < .001).

## Discussion

### Grassland site, catenary position, and soil depth drive microbial community structure

The fungal and bacterial communities were sampled from two grasslands relatively similar in climate and soil properties but that differed in land-use history, particularly with regard to time and management since cessation of intensive agriculture. This legacy was expressed in the vegetation characteristics, i.e. root biomass, plant species richness, composition of plant functional groups, and proximity to the forest border. Differences were found in both fungal and bacterial community compositions across grasslands, elevations, and soil depths, with grasslands—likely derived from land use—emerging as the strongest control in the RDA. This corroborates the first hypothesis that these three factors are important drivers of microbial community structure. Topography can affect microbial communities by influencing water or nutrient availability directly through slope runoff or indirectly through plant growth (Zhang et al. 2013). While soil moisture was negatively correlated with microbial abundance in our sites, catenary position had a significant effect on microbial community composition, but not abundance. This suggests that certain microbial taxa might have been particularly sensitive even to slight variations in topography, and highlights the importance of integrating measures of landscape heterogeneity with land use for a comprehensive understanding of nutrient availability and limitations on soil microbial communities. Moreover, while microbial communities tend to be more homogeneous in deeper soil layers (Eilers et al. 2012), it is important to recognize that this pattern is influenced by both land use (van Leeuwen et al. 2017) and topography. Therefore, it becomes essential to also consider soil depth as an additional dimension in the environmental gradient that contributes to the structure and function of microbial communities (Eilers et al. 2012). The integration of land use, topography, and soil depth will allow for a better understanding of the scales that determine microbial dynamics and spatial variation in soils. However, these three factors explained twice the variation for bacteria than for fungi. This could partly be explained by the fact that fungal hyphae can form networks that cover extensive areas (Dahlberg and Stenlid 1990, Genney et al. 2006), grow across the depths and elevations considered, and have longer life spans compared to bacteria. This means that despite the pooling of the soil core samples to attain representative samples at the plot scale, the lower explained variation of fungal community composition could be due to fungal communities being more spatially uniform than bacterial communities because of their specific biotic characteristics. In addition, the higher number of bacterial OTUs, resulting also from the different sequencing methods used, improves prediction potential for bacteria.

### Partial evidence for plant–soil microbial community feedback

In further support of our first hypothesis, the variation in microbial community composition was explained by plant variables (root biomass, species richness, proportion of grasses and forbs, and distance to the forest edge) for both fungi and bacteria, but also by some of the edaphic variables. Despite previous studies (Dassen et al. 2017, Liu et al. 2020) suggesting a stronger link between vegetation and fungal community composition compared to bacteria, the total explained variation for fungi remained lower than for bacteria. The distance to the closest tree and the number of plant species were the drivers explaining most of the variation for both fungal and bacterial communities, and were also the only two drivers significant across all depths. This suggests an important role of plant species identity and plant species richness in shaping both fungal and bacterial communities (Dassen et al. 2017) even in deeper soil layers. In our study, plant species richness was determined by observations aboveground. Previous work has shown that plant identification through metabarcoding from root samples can reveal higher species richness compared to the values obtained by conventional plant inventories aboveground (Hiiesalu et al. 2014), which could improve the explanatory power of analyses of linkages between plant and microbial communities. Further, soil pH is commonly a strong driver of microbial communities (Pellissier et al. 2014, Cao et al. 2017, Hao et al. 2021). Here, since pH varied with soil depth, but not considerably across grasslands or catenary positions, and was significantly correlated with SOM, it did not emerge as a significant predictor for bacteria and did not contribute to explaining the fungal community composition in this study.

The higher abundance of ectomycorrhizal fungi in Ämtvik was likely driven by the proximity to the forest edge (6–20 m on average in Ämtvik, compared to 50–90 m in Tovetorp), as they are mostly associated to trees (Dickie and Reich 2005). While plant–mycorrhizal associations may directly affect plant species distribution by favoring taxa that are connected to the hyphal network (Ettema and Wardle 2002), in this case, the proximity of trees was negatively correlated with grassland plant species richness, possibly due to shading and competition with tree roots. This may partly explain why arbuscular mycorrhizal fungi (typically associated with grassland species) were predominantly found in the more open locations, as was also observed by Moora et al. (2014). In addition, the covariation between arbuscular mycorrhizal fungi communities and plant communities decreases along a succession gradient from open grassland to forest (Neuenkamp et al. 2018). Therefore, it is not surprising that the distance from the forest edge emerges as an important driver of microbial community composition at our sites, suggesting that future studies on grassland biodiversity should integrate the patch size of grasslands and the proximity to forests in complex landscapes using spatial analyses also when observing soil microbial communities.

### Depth effects on microbial abundance and diversity

Fungal and bacterial abundances decreased with depth, indicating a decline in microbial biomass and biological activity below the rooting zone, and thus confirming part of our second hypothesis. Such decrease has also been shown by other authors (Fierer et al. 2003, Xu et al. 2013, Hao et al. 2021), and has been attributed to a reduction of the availability of C sources with depth (Fierer et al. 2003). This interpretation is supported by our results, which showed a similar reduction in root biomass, soil C, and SOM with soil depth. Fungal abundance decreased with depth in all functional groups except for arbuscular mycorrhiza and ectomycorrhiza, which were unrelated to depth. This finding aligns with Schlatter et al. (2018) and suggests that the drivers of community composition of mycorrhizal fungi are distinct from those of other functional groups and might instead be more linked to specific plant host abundance and rooting depth.

Despite a significant decline in species richness, only bacterial Shannon diversity decreased slightly with depth, as in Eilers et al. (2012), thus partly rejecting our second hypothesis. It is possible that the relatively high diversity in deeper soil layers is a result of more scarce resources promoting coexistence of microbial foraging strategies, in contrast to richer surface conditions where fewer more competitive species can dominate.

### Plant functional group composition is a better predictor of microbial diversity than plant diversity

Bacterial diversity correlated negatively only to soil moisture, while fungal diversity showed no significant correlations to edaphic or vegetation factors. This partly rejects our third hypothesis of a general correlation between microbial diversity and plant diversity. It also indicates that the drivers of fungal and bacterial diversity are separate from the drivers of microbial abundance. Previous studies, however, have shown that microbial diversity in grasslands may require larger-scale sampling to be accurately mapped (Pellissier et al. 2014), and that the interactions within microbial and between microbial and plant communities can be affected by the presence and ecosystem functions of soil macrofauna (Wardle et al. 2004), which was not included in this study. In addition, the low mycorrhizal abundance, particularly of arbuscular mycorrhizae, was likely due to methodological limitations. First, the microbial DNA was extracted from bulk soil and not from roots. Second, the primer gITS7 does not cover all arbuscular mycorrhizal fungi (Cheeke et al. 2021), and more specific primers would be needed to capture that community.

Finally, our study highlights the significance of considering different microbial functional groups and their correlations with environmental variables when examining ecosystem functioning and community response to environmental change, as previously suggested by Frac et al. (2018). Consistent with earlier studies (Wardle et al. 2004, Hiiesalu et al. 2014), we found a strong association between mycorrhizal diversity and vegetation properties, likely influenced by forest edge effects. Additionally, saprotroph diversity was correlated with plant diversity and various measures of plant community composition, as also observed by Strecker et al. (2015), indicating a potential impact of plant functional groups or species composition on litter quality (e.g. lignin and N contents). These findings suggest that correlations between vegetation and microbial diversity differ among microbial functional groups, and imply that plant functional groups might serve as better predictors of microbial diversity than plant diversity, as previously demonstrated for saprotrophic fungi (Francioli et al. 2021).

## Conclusion

Fungal and bacterial community composition differed between grasslands (potentially linked to differences in vegetation and land-use history), elevations, and soil depths. The tested edaphic and plant variables generally explained twice as much of the community variation for bacteria than for fungi. This difference could be due to the scale at which the drivers and the communities are derived, or because other drivers, such as biotic interactions among fungi and between fungi and plant species, are relatively more important for fungi than for bacteria. By characterizing microbial communities across elevation positions under the same climate and soil, and along the soil depth profile, we identified resource availability (root biomass and soil C and N contents), soil moisture, plant functional group composition, and species richness as the main drivers of microbial abundance and diversity. Microbial abundance and bacterial diversity decreased with depth, but fungal diversity remained stable. While there was no general correlation between microbial diversity and plant diversity, diversity within specific microbial groups (mycorrhizal and saprotrophic fungi) was correlated with the occurrence of some plant functional groups, indicating interactions between ecosystem functions aboveground and belowground. Finally, integration of land use, topography, and soil depth will allow for a better understanding of the scales that determine microbial dynamics and spatial variation in grassland soils.

## Funding

This research was funded by the Bolin Centre for Climate Research at Stockholm University and by the Carl Mannerfelt Foundation. S.M. received funding from the European Research Council (ERC) under the European Union’s Horizon 2020 research and innovation programme (grant agreement no 101001608). P.F. was supported by the Swedish Research Council FORMAS (2016-01107) and K.E.C. by FORMAS (2020-01110).

## Supplementary Material

fiad080_Supplemental_File
