# Supplementary material for: Vegetation, topography, and soil depth drive microbial community structure in two Swedish grasslands
Source: FEMS Microbiol Ecol. 2023 Jul 20;99(8):fiad080. doi: 10.1093/femsec/fiad080 (PMC10370287; doi:10.1093/femsec/fiad080)
Supplement: fiad080_Supplemental_File [file fiad080_supplemental_file.docx]

Supplementary material

## Tables

**Table S1**. **Geographical coordinates**

Study location coordinates for the two grasslands in south-central Sweden, Tovetorp and Ämtvik.

| **Location** | **Coordinates** |
| --- | --- |
| Tovetorp High | 58.945875 N 17.149324 E |
| Tovetorp Low | 58.945221 N 17.149464 E |
| Ämtvik High | 58.959972 N 17.093950 E |
| Ämtvik Low | 58.958709 N 17.094144 E |

**Table S2.** **Redundancy analyses**

Redundancy analyses (RDA) of fungal (a, b) and bacterial (c, d) communities in soil profiles at two grassland sites with two elevations in south-central Sweden. (a, b) RDAs of all identified fungal species (780) in soil sampled at 0-10, 10-20, 20-30 and 40-50 cm (n=48) testing categorical treatments (a) and edaphic and plant variables (b). (c, d) RDAs of bacterial taxa (7115) for the same four soil depths (n=48). The effect of grassland, elevation and depth was tested by restricted Monte Carlo permutations accounting for dependency of communities in soil depths from the same plot, using forward selection of explanatory variables (False discovery rate correction). Sequencing depth was included as a covariate.

1. RDA of all identified fungi testing categorical treatments (Fig. S2)

| **Main effect** | **F** | | | **P (adj.)** |
| --- | --- | --- | --- | --- |
| *Global test of grassland, elevation and depth:* |  | | |  |
| First axis | 6.5 | | | 0.002 |
| All axes | 4.3 | | | 0.002 |
| Sum of partial variation: | 41973.6 | | |  |
| Explained variation (adj.): | 17.6% | | |  |
| *Selected explanatory variables:* | |  |  | |
| Grassland | 6.7 | | | 0.0025 |
| Elevation | 3.4 | | | 0.002 |
| Depth | 2.2 | | | 0.002 |
| Sum of partial variation: | 41973.6 | | |  |
| Explained variation (adj.): | 17.4% | | |  |
| Covariate: | Sequencing depth | | |  |

1. RDA of all identified fungi testing edaphic and plant variables (Fig. S2)

| **Main effect** | **F** | | | **P (adj.)** |
| --- | --- | --- | --- | --- |
| *Global test of edaphic and plant variables:* |  | | |  |
| First axis | 5.9 | | | 0.002 |
| All axes | 3.1 | | | 0.002 |
| Sum of partial variation: | 41973.6 | | |  |
| Explained variation (adj.): | 26.5% | | |  |
| *Selected explanatory variables:* | |  |  | |
| Closest tree (m) | 6.4 | | | 0.0053 |
| Plant species richness (-) | 4.4 | | | 0.004 |
| Soil moisture (y) | 2.4 | | | 0.008 |
| Forb biomass (%) | 2.2 | | | 0.004 |
| Grass biomass (%) | 2.1 | | | 0.0032 |
| Aboveground biomass (g/m^2^) | 1.6 | | | 0.014 |
| Root biomass (g) | 1.6 | | | 0.005 |
| SOM (%) | 1.4 | | | 0.028 |
| Sum of partial variation: | 41973.6 | | |  |
| Explained variation (adj.): | 26.5% | | |  |
| Covariate: | Sequencing depth | | |  |

1. RDA of bacterial taxa testing categorical treatments (Fig. S2)

| **Main effect** | **F** | | | **P (adj.)** |
| --- | --- | --- | --- | --- |
| *Global test of grassland, elevation and depth:* |  | | |  |
| First axis | 12.3 | | | 0.002 |
| All axes | 10.8 | | | 0.002 |
| Sum of partial variation: | 46586.3 | | |  |
| Explained variation (adj.): | 39.1% | | |  |
| *Selected explanatory variables:* | |  |  | |
| Elevation | 14.1 | | | 0.0033 |
| Depth | 8.1 | | | 0.0025 |
| Grassland | 5.3 | | | 0.008 |
| Sum of partial variation: | 46586.3 | | |  |
| Explained variation (adj.): | 39.1% | | |  |
| Covariate: | Sequencing depth | | |  |

1. RDA of bacterial taxa testing edaphic and plant variables (Fig. S2)

| **Main effect** | **F** | | | **P (adj.)** |
| --- | --- | --- | --- | --- |
| *Global test of edaphic and plant variables:* |  | | |  |
| First axis | 10.6 | | | 0.004 |
| All axes | 7.5 | | | 0.002 |
| Sum of partial variation: | 46586.3 | | |  |
| Explained variation (adj.): | 56.1% | | |  |
| *Selected explanatory variables:* | |  |  | |
| Closest tree (m) | 11.9 | | | 0.002 |
| Plant species richness (-) | 10.3 | | | 0.002 |
| Soil moisture (y) | 8.3 | | | 0.002 |
| Soil N (μg/mg) | 7.9 | | | 0.002 |
| Root biomass (g) | 2.5 | | | 0.004 |
| Forbs (%) | 2.4 | | | 0.01 |
| Grasses (%) | 2.3 | | | 0.012 |
| Soil C (μg/mg) | 2.0 | | | 0.0028 |
| Sum of partial variation: | 46586.3 | | |  |
| Explained variation (adj.): | 56.3% | | |  |
| Covariate: | Sequencing depth | | |  |

**Table S3.** **Analysis of Similarity**

Correlation coefficient and P values for the Analysis of Similarity (ANOSIM) between fungal and bacterial dissimilarity matrices, respectively, and the categories grassland, elevation and soil depth.

| **Category** | **Fungi** | | **Bacteria** | |
| --- | --- | --- | --- | --- |
|  | **corr** | **P** | **corr** | **P** |
| Depth | 0.3764 | 1e-04 | 0.3398 | < 0.001 |
| Grassland | 0.2092 | 1e-04 | 0.176 | < 0.001 |
| Elevation | 0.1171 | 0.0034 | 0.1007 | 0.0075 |

**Table S4. Mvabund analyses**

Deviance values and P values for mvabund analyses of (A) fungal functional groups, (B) fungal divisions and (C) bacterial phyla. The abundance of DNA counts was compared between grasslands, elevations and depths for all groups. Significant relationships marked with (*) at P < 0.05.

| **A) Fungal functional groups** |  | **Grassland** | **Elevation** | **Depth** |
| --- | --- | --- | --- | --- |
| Unknown | **Dev** | 0.779 | 0.87 | 24.468 |
|  | **Pr(>Dev)** | 0.856 | 0.884 | 0.014* |
| Animal parasite | **Dev** | 0.001 | 0.748 | 39.043 |
|  | **Pr(>Dev)** | 0.998 | 0.903 | 0.001* |
| Mycoparasite | **Dev** | 2.401 | 0.318 | 27.397 |
|  | **Pr(>Dev)** | 0.642 | 0.962 | 0.009* |
| Plant pathogen | **Dev** | 0 | 0.053 | 44.39 |
|  | **Pr(>Dev)** | 0.998 | 0.985 | 0.001* |
| Endophyte | **Dev** | 3.472 | 1.994 | 21.098 |
|  | **Pr(>Dev)** | 0.624 | 0.677 | 0.025* |
| Lichenized and lichen parasite | **Dev** | 0.167 | 0.033 | 22.663 |
|  | **Pr(>Dev)** | 0.917 | 0.985 | 0.024* |
| Wood saprotroph | **Dev** | 0.421 | 0.981 | 29.852 |
|  | **Pr(>Dev)** | 0.907 | 0.854 | 0.007* |
| Litter saprotroph | **Dev** | 0.796 | 0.1 | 19.509 |
|  | **Pr(>Dev)** | 0.856 | 0.985 | 0.030* |
| Dung saprotroph | **Dev** | 2.178 | 3.419 | 17.836 |
|  | **Pr(>Dev)** | 0.672 | 0.523 | 0.038* |
| Soil saprotroph | **Dev** | 0.561 | 0.265 | 32.405 |
|  | **Pr(>Dev)** | 0.886 | 0.962 | 0.003* |
| Undefined saprotroph | **Dev** | 0.313 | 0.634 | 28.702 |
|  | **Pr(>Dev)** | 0.917 | 0.919 | 0.008* |
| Arbuscular mycorrhiza | **Dev** | 4.408 | 9.406 | 4.137 |
|  | **Pr(>Dev)** | 0.511 | 0.085 | 0.723 |
| Ectomycorrhiza | **Dev** | 18.989 | 0.359 | 2.727 |
|  | **Pr(>Dev)** | 0.005* | 0.962 | 0.723 |

| **B) Fungal phyla** |  | **Grassland** | **Elevation** | **Depth** |
| --- | --- | --- | --- | --- |
| Ascomycota | **Dev** | 0.002 | 0.115 | 32.76 |
|  | **Pr(>Dev)** | 0.962 | 0.954 | 0.002* |
| Basidiomycota | **Dev** | 0.036 | 0.053 | 23.558 |
|  | **Pr(>Dev)** | 0.942 | 0.968 | 0.007* |
| Calcarisporiellomycota | **Dev** | 5.98 | 2.513 | 5.517 |
|  | **Pr(>Dev)** | 0.134 | 0.496 | 0.624 |
| Chytridiomycota | **Dev** | 0.538 | 3.331 | 16.769 |
|  | **Pr(>Dev)** | 0.806 | 0.448 | 0.027* |
| Glomeromycota | **Dev** | 2.107 | 3.099 | 9.139 |
|  | **Pr(>Dev)** | 0.486 | 0.460 | 0.439 |
| Mortierellomycota | **Dev** | 0.437 | 2.775 | 29.023 |
|  | **Pr(>Dev)** | 0.806 | 0.470 | 0.005* |
| Mucoromycota | **Dev** | 6.825 | 1.253 | 26.849 |
|  | **Pr(>Dev)** | 0.125 | 0.631 | 0.005* |
| Zygomycota | **Dev** | 3.599 | 0.023 | 8.02 |
|  | **Pr(>Dev)** | 0.350 | 0.968 | 0.534 |
| unclassified.fungi | **Dev** | 7.971 | 0.476 | 27.615 |
|  | **Pr(>Dev)** | 0.089 | 0.846 | 0.005* |

| **C) Bacteria phyla** |  | **Grassland** | **Elevation** | **Depth** |
| --- | --- | --- | --- | --- |
| Acidobacteriota | **Dev** | 0.762 | 0.116 | 27.984 |
|  | **Pr(>Dev)** | 0.971 | 1.000 | 0.014* |
| Actinobacteriota | **Dev** | 0.947 | 0.001 | 35.527 |
|  | **Pr(>Dev)** | 0.943 | 1.000 | 0.001* |
| Armatimonadota | **Dev** | 9.463 | 1.001 | 32.789 |
|  | **Pr(>Dev)** | 0.067 | 0.991 | 0.004* |
| Bacteroidota | **Dev** | 0.004 | 0.163 | 31.344 |
|  | **Pr(>Dev)** | 1.000 | 1.000 | 0.005* |
| Bdellovibrionota | **Dev** | 2.087 | 0.067 | 40.265 |
|  | **Pr(>Dev)** | 0.789 | 1.000 | 0.001* |
| Chloroflexi | **Dev** | 4.634 | 1.114 | 23.979 |
|  | **Pr(>Dev)** | 0.385 | 0.985 | 0.042* |
| Crenarchaeota | **Dev** | 7.366 | 0.513 | 15.924 |
|  | **Pr(>Dev)** | 0.170 | 0.999 | 0.229 |
| Cyanobacteria | **Dev** | 4.729 | 0.956 | 31.652 |
|  | **Pr(>Dev)** | 0.385 | 0.992 | 0.004* |
| Dadabacteria | **Dev** | 6.244 | 0.135 | 8.9 |
|  | **Pr(>Dev)** | 0.243 | 1.000 | 0.688 |
| Deinococcota | **Dev** | 0.099 | 0.005 | 7.277 |
|  | **Pr(>Dev)** | 1.000 | 1.000 | 0.741 |
| Dependentiae | **Dev** | 1.374 | 0.079 | 21.234 |
|  | **Pr(>Dev)** | 0.900 | 1.000 | 0.076 |
| Desulfobacterota | **Dev** | 3.018 | 0.284 | 17.077 |
|  | **Pr(>Dev)** | 0.665 | 1.000 | 0.192 |
| Elusimicrobiota | **Dev** | 1.911 | 0.28 | 19.845 |
|  | **Pr(>Dev)** | 0.811 | 1.000 | 0.099 |
| Entotheonellaeota | **Dev** | 1.093 | 0.775 | 14.291 |
|  | **Pr(>Dev)** | 0.924 | 0.996 | 0.297 |
| Euryarchaeota | **Dev** | 6.412 | 0.363 | 14.578 |
|  | **Pr(>Dev)** | 0.235 | 0.999 | 0.294 |
| Fibrobacterota | **Dev** | 0.403 | 0.157 | 26.546 |
|  | **Pr(>Dev)** | 0.996 | 1.000 | 0.021* |
| Firmicutes | **Dev** | 1.483 | 0.153 | 32.178 |
|  | **Pr(>Dev)** | 0.897 | 1.000 | 0.004* |
| Fusobacteriota | **Dev** | 1.494 | 1.624 | 8.387 |
|  | **Pr(>Dev)** | 0.897 | 0.956 | 0.688 |
| GAL15 | **Dev** | 0.004 | 7.384 | 6.242 |
|  | **Pr(>Dev)** | 1.000 | 0.193 | 0.760 |
| Gemmatimonadota | **Dev** | 7.233 | 0.005 | 24.058 |
|  | **Pr(>Dev)** | 0.175 | 1.000 | 0.041* |
| Halanaerobiaeota | **Dev** | 7.762 | 0.044 | 9.482 |
|  | **Pr(>Dev)** | 0.134 | 1.000 | 0.658 |
| Latescibacterota | **Dev** | 6.126 | 1.617 | 21.559 |
|  | **Pr(>Dev)** | 0.243 | 0.956 | 0.072 |
| MBNT15 | **Dev** | 4.892 | 0.043 | 10.191 |
|  | **Pr(>Dev)** | 0.385 | 1.000 | 0.583 |
| Methylomirabilota | **Dev** | 5.452 | 0.158 | 6.541 |
|  | **Pr(>Dev)** | 0.314 | 1.000 | 0.760 |
| Unclassified.bacteria | **Dev** | 0.175 | 0.485 | 20.427 |
|  | **Pr(>Dev)** | 1.000 | 0.999 | 0.087 |
| NB1.j | **Dev** | 0.21 | 0.933 | 2.938 |
|  | **Pr(>Dev)** | 1.000 | 0.992 | 0.851 |
| Nitrospirota | **Dev** | 4.218 | 1.793 | 6.381 |
|  | **Pr(>Dev)** | 0.405 | 0.927 | 0.760 |
| Patescibacteria | **Dev** | 0.082 | 0.058 | 34.567 |
|  | **Pr(>Dev)** | 1.000 | 1.000 | 0.002* |
| Planctomycetota | **Dev** | 0.434 | 0.09 | 32.907 |
|  | **Pr(>Dev)** | 0.995 | 1.000 | 0.004* |
| Proteobacteria | **Dev** | 0.029 | 0.119 | 33.887 |
|  | **Pr(>Dev)** | 1.000 | 1.000 | 0.002* |
| RCP2.54 | **Dev** | 0.015 | 7.803 | 17.128 |
|  | **Pr(>Dev)** | 1.000 | 0.160 | 0.191 |
| SAR324.cla.Mar.gr.B. | **Dev** | 1.816 | 0.203 | 14.122 |
|  | **Pr(>Dev)** | 0.811 | 1.000 | 0.297 |
| Spirochaetota | **Dev** | 1.898 | 0.521 | 8.548 |
|  | **Pr(>Dev)** | 0.811 | 0.999 | 0.688 |
| Sumerlaeota | **Dev** | 0.077 | 0.037 | 30.083 |
|  | **Pr(>Dev)** | 1.000 | 1.000 | 0.007* |
| Verrucomicrobiota | **Dev** | 0.083 | 0.193 | 33.297 |
|  | **Pr(>Dev)** | 1.000 | 1.000 | 0.004* |
| WPS.2 | **Dev** | 1.102 | 4.579 | 7.265 |
|  | **Pr(>Dev)** | 0.924 | 0.471 | 0.741 |
| WS2 | **Dev** | 0.036 | 1.587 | 3.056 |
|  | **Pr(>Dev)** | 1.000 | 0.956 | 0.851 |

**Table S5. Correlation tests of microbial diversity and environmental variables**

Correlation coefficients for species richness and Shannon diversity of (A) bacteria and (B) fungi, including phyla, divisions and major fungal functional groups. Only statistically significant correlations are reported (P < 0.05).

| **(A)** |  | **corr** | **P** |
| --- | --- | --- | --- |
| sp. richness bacteria | SOM | 0.3322 | 0.0225 |
| sp. richness bacteria | soil moisture | -0.4728 | < 0.001 |
| sp. richness bacteria | total soil C | 0.4885 | < 0.001 |
| sp. richness bacteria | root biomass | 0.3063 | 0.0362 |
| sp. richness bacteria | total soil N | 0.3588 | 0.0132 |
| sp. richness bacteria | Soil pH | -0.3735 | 0.009 |
| shannon (H’) bacteria | soil moisture | -0.4232 | 0.003 |
| shannon (H’) Acidobacteriota | % grasses (biomass) | -0.2950 | 0.0441 |
| shannon (H’) Acidobacteriota | % legumes (biomass) | 0.3510 | 0.0156 |
| shannon (H’) Acidobacteriota | forbs (sp. richness) | 0.4216 | 0.0032 |
| shannon (H’) Acidobacteriota | plant species richness | 0.3974 | 0.0057 |
| shannon (H’) Acidobacteriota | soil moisture | -0.5459 | < 0.001 |
| shannon (H’) Acidobacteriota | total soil C | 0.4260 | 0.0028 |
| shannon (H’) Acidobacteriota | total soil N | 0.3562 | 0.0139 |
| shannon (H’) Acidobacteriota | root biomass | 0.3920 | 0.0064 |
| shannon (H’) Acidobacteriota | Soil pH | -0.395 | 0.006 |
| shannon (H’) Actinobacteriota | aboveground plant biomass | -0.5357 | < 0.001 |
| shannon (H’) Actinobacteriota | plant species richness | 0.4202 | 0.0033 |
| shannon (H’) Actinobacteriota | plant diversity (Shannon i) | 0.4284 | 0.0027 |
| shannon (H’) Actinobacteriota | forbs (sp. richness) | 0.3328 | 0.0223 |
| shannon (H’) Actinobacteriota | Soil pH | -0.2968 | 0.0427 |
| shannon (H’) Chloroflexi | distance from trees | 0.5637 | < 0.001 |
| shannon (H’) Chloroflexi | plant species richness | -0.4121 | 0.0040 |
| shannon (H’) Chloroflexi | % forbs (biomass) | -0.5166 | < 0.001 |
| shannon (H’) Chloroflexi | % legumes (biomass) | -0.4107 | 0.0041 |
| shannon (H’) Chloroflexi | forbs (sp. richness) | -0.4838 | 0.0006 |
| shannon (H’) Proteobacteria | soil moisture | -0.3161 | 0.0304 |
| shannon (H’) Verrucomicrobiota | distance from trees | -0.2914 | 0.0469 |
| shannon (H’) Verrucomicrobiota | grasses (sp. richness) | -0.3033 | 0.0383 |
| sp. richness Acidobacteriota | % legumes (biomass) | 0.4387 | 0.0026 |
| sp. richness Acidobacteriota | forbs (sp. richness) | 0.4074 | 0.0055 |
| sp. richness Actinobacteriota | aboveground plant biomass | -0.3257 | 0.0255 |
| sp. richness Actinobacteriota | SOM | 0.2984 | 0.0416 |
| sp. richness Actinobacteriota | soil moisture | -0.4707 | < 0.001 |
| sp. richness Actinobacteriota | total soil C | 0.3633 | 0.0121 |
| sp. richness Actinobacteriota | total soil N | 0.7774 | < 0.001 |
| sp. richness Actinobacteriota | plant diversity (Shannon i) | 0.3188 | 0.0290 |
| sp. richness Actinobacteriota | root biomass | 0.3870 | 0.0072 |
| sp. richness Chloroflexi | distance from trees | 0.6134 | < 0.001 |
| sp. richness Chloroflexi | % forbs (biomass) | -0.6714 | < 0.001 |
| sp. richness Chloroflexi | plant diversity (Shannon i) | -0.4080 | 0.0044 |
| sp. richness Chloroflexi | % legumes (biomass) | -0.5046 | 0.0003 |
| sp. richness Chloroflexi | plant species richness | -0.5326 | 0.001 |
| sp. richness Chloroflexi | forbs (sp. richness) | -0.6099 | < 0.001 |
| sp. richness Proteobacteria | % legumes (biomass) | 0.2923 | 0.0462 |
| sp. richness Proteobacteria | forbs (sp. richness) | 0.2941 | 0.0448 |
| sp. richness Proteobacteria | SOM | 0.2930 | 0.0457 |
| sp. richness Proteobacteria | soil moisture | -0.5816 | < 0.001 |
| sp. richness Proteobacteria | total soil C | 0.5104 | < 0.001 |
| sp. richness Proteobacteria | total soil N | 0.4246 | 0.0029 |
| sp. richness Proteobacteria | root biomass | 0.4695 | 0.0009 |
| sp. richness Proteobacteria | Soil pH | -0.4077 | 0.004 |
| sp. richness Verrucomicrobiota | distance from trees | -0.3958 | 0.0059 |
| sp. richness Verrucomicrobiota | % forbs (biomass) | 0.3492 | 0.0161 |
| sp. richness Verrucomicrobiota | % legumes (biomass) | 0.3895 | 0.0068 |
| sp. richness Verrucomicrobiota | grasses (sp. richness) | -0.3650 | 0.0116 |
| sp. richness Verrucomicrobiota | forbs (sp. richness) | 0.3532 | 0.0149 |
| sp. richness Verrucomicrobiota | SOM | 0.3370 | 0.0205 |
| sp. richness Verrucomicrobiota | soil moisture | -0.5196 | < 0.001 |
| sp. richness Verrucomicrobiota | total soil C | 0.5754 | < 0.001 |
| sp. richness Verrucomicrobiota | total soil N | 0.4813 | < 0.001 |
| sp. richness Verrucomicrobiota | root biomass | 0.3733 | 0.0098 |
| sp. richness Verrucomicrobiota | Soil pH | -0.4709 | < 0.001 |

| **(B)** |  | **corr** | **P** |
| --- | --- | --- | --- |
| Shannon (H’) Ascomycota | SOM | 0.4121 | 0.0040 |
| Shannon (H’) Ascomycota | root biomass | 0.3980 | 0.0056 |
| Shannon (H’) Ascomycota | soil moisture | -0.4537 | 0.0014 |
| Shannon (H’) Ascomycota | total soil C | 0.5347 | < 0.001 |
| Shannon (H’) Ascomycota | total soil N | 0.5213 | < 0.001 |
| Shannon (H’) Ascomycota | Soil pH | -0.555 | < 0.001 |
| Shannon (H’) Basidiomycota | % forbs (biomass) | 0.4253 | 0.0029 |
| Shannon (H’) Dung saprotrophs | soil moisture | -0.3090 | 0.0346 |
| Shannon (H’) Litter saprotrophs | root biomass | 0.3294 | 0.0238 |
| Shannon (H’) Litter saprotrophs | soil moisture | -0.3604 | 0.0128 |
| Shannon (H’) Litter saprotrophs | total soil C | 0.4316 | 0.0025 |
| Shannon (H’) Litter saprotrophs | total soil N | 0.3913 | 0.0065 |
| Shannon (H’) Litter saprotrophs | grasses (sp. richness) | -0.3806 | 0.0083 |
| Shannon (H’) Litter saprotrophs | Soil pH | -0.4932 | < 0.001 |
| Shannon (H’) Mycorrhizal fungi | aboveground plant biomass | -0.3384 | 0.02 |
| Shannon (H’) Mycorrhizal fungi | distance from trees | -0.6498 | < 0.001 |
| Shannon (H’) Mycorrhizal fungi | forbs (sp. richness) | 0.7551 | < 0.001 |
| Shannon (H’) Mycorrhizal fungi | % forbs (biomass) | 0.5950 | < 0.001 |
| Shannon (H’) Mycorrhizal fungi | total soil N | -0.3237 | 0.0265 |
| Shannon (H’) Mycorrhizal fungi | % legumes (biomass) | 0.6302 | < 0.001 |
| Shannon (H’) Mycorrhizal fungi | plant diversity (Shannon i) | 0.4599 | < 0.001 |
| Shannon (H’) Mycorrhizal fungi | plant species richness | 0.7074 | < 0.001 |
| Shannon (H’) Pathogen fungi | soil moisture | -0.2906 | 0.0475 |
| Shannon (H’) Saprotrophs | % forbs (biomass) | 0.3424 | 0.0185 |
| Shannon (H’) Soil saprotrophs | distance from trees | -0.7235 | < 0.001 |
| Shannon (H’) Soil saprotrophs | forbs (sp. richness) | 0.6234 | < 0.001 |
| Shannon (H’) Soil saprotrophs | % forbs (biomass) | 0.7750 | < 0.001 |
| Shannon (H’) Soil saprotrophs | plant diversity (Shannon i) | 0.4156 | 0.0037 |
| Shannon (H’) Soil saprotrophs | plant species richness | 0.5129 | <0.001 |
| Shannon (H’) Soil saprotrophs | % legumes (biomass) | 0.5626 | < 0.001 |
| Shannon (H’) Undefined saprotrophs | soil moisture | -0.3549 | 0.0144 |
| Shannon (H’) Undefined saprotrophs | Soil pH | -0.4004 | 0.0053 |
| Shannon (H’) Wood saprotrophs | aboveground plant biomass | 0.3059 | 0.0365 |
| Shannon (H’) Wood saprotrophs | Soil pH | -0.4064 | 0.0046 |
| sp. richness fungi | SOM | 0.3706 | 0.0103 |
| sp. richness fungi | root biomass | 0.3170 | 0.0299 |
| sp. richness fungi | soil moisture | -0.5935 | < 0.001 |
| sp. richness fungi | total soil C | 0.4117 | 0.0040 |
| sp. richness fungi | soil pH | -0.5738 | < 0.001 |
| sp. richness Ascomycota | SOM | 0.4469 | 0.0016 |
| sp. richness Ascomycota | root biomass | 0.4536 | 0.0014 |
| sp. richness Ascomycota | soil moisture | -0.6386 | < 0.001 |
| sp. richness Ascomycota | total soil C | 0.4925 | < 0.001 |
| sp. richness Ascomycota | total soil N | 0.4639 | 0.0010 |
| sp. richness Ascomycota | soil pH | -0.6207 | < 0.001 |
| sp. richness Basidiomycetes | distance from trees | -0.4316 | 0.0025 |
| sp. richness Basidiomycetes | forbs (sp. richness) | 0.3813 | 0.0082 |
| sp. richness Basidiomycetes | plant species richness | 0.3118 | 0.0329 |
| sp. richness Basidiomycetes | % forbs (biomass) | 0.4678 | < 0.001 |
| sp. richness Basidiomycetes | % legumes (biomass) | 0.4166 | 0.0036 |
| sp. richness Basidiomycetes | soil pH | -0.3047 | 0.0372 |
| sp. richness Dung saprotrophs | soil moisture | -0.3152 | 0.0309 |
| sp. richness Litter saprotrophs | SOM | 0.4664 | 0.0010 |
| sp. richness Litter saprotrophs | root biomass | 0.4562 | 0.0013 |
| sp. richness Litter saprotrophs | soil moisture | -0.6053 | < 0.001 |
| sp. richness Litter saprotrophs | total soil C | 0.6684 | < 0.001 |
| sp. richness Litter saprotrophs | total soil N | 0.6047 | < 0.001 |
| sp. richness Litter saprotrophs | grasses (sp. richness) | -0.3637 | 0.0120 |
| sp. richness Litter saprotrophs | soil pH | -0.6484 | < 0.001 |
| sp. richness Mycorrhizal fungi | aboveground plant biomass | -0.4559 | 0.0011 |
| sp. richness Mycorrhizal fungi | distance from trees | -0.6789 | < 0.001 |
| sp. richness Mycorrhizal fungi | forbs (sp. richness) | 0.8206 | < 0.001 |
| sp. richness Mycorrhizal fungi | % forbs (biomass) | 0.5810 | < 0.001 |
| sp. richness Mycorrhizal fungi | % legumes (biomass) | 0.6565 | < 0.001 |
| sp. richness Mycorrhizal fungi | plant diversity (Shannon i) | 0.4454 | 0.0017 |
| sp. richness Mycorrhizal fungi | plant species richness | 0.7721 | <0.001 |
| sp. richness Pathogen fungi | soil moisture | -0.3266 | 0.0251 |
| sp. richness Saprotrophs | distance from trees | -0.4662 | 0.0010 |
| sp. richness Saprotrophs | soil moisture | -0.4998 | 0.0003 |
| sp. richness Saprotrophs | total soil C | 0.3974 | 0.0057 |
| sp. richness Saprotrophs | total soil N | 0.2935 | 0.0452 |
| sp. richness Saprotrophs | forbs (sp. richness) | 0.3376 | 0.0203 |
| sp. richness Saprotrophs | grasses (sp. richness) | -0.3130 | 0.0322 |
| sp. richness Saprotrophs | % forbs (biomass) | 0.4539 | 0.0014 |
| sp. richness Saprotrophs | % legumes (biomass) | 0.3903 | 0.0067 |
| sp. richness Saprotrophs | soil pH | -0.4981 | < 0.001 |
| sp. richness Soil saprotrophs | aboveground plant biomass | -0.3323 | 0.0225 |
| sp. richness Soil saprotrophs | distance from trees | -0.7255 | < 0.001 |
| sp. richness Soil saprotrophs | forbs (sp. richness) | 0.6820 | < 0.001 |
| sp. richness Soil saprotrophs | grasses (sp. richness) | -0.3389 | 0.0198 |
| sp. richness Soil saprotrophs | % forbs (biomass) | 0.6736 | < 0.001 |
| sp. richness Soil saprotrophs | plant species richness | 0.5828 | <0.001 |
| sp. richness Soil saprotrophs | plant diversity (Shannon i) | 0.3408 | 0.0191 |
| sp. richness Soil saprotrophs | % legumes (biomass) | 0.6169 | < 0.001 |
| sp. richness Undefined saprotrophs | aboveground plant biomass | 0.3037 | 0.0380 |
| sp. richness Undefined saprotrophs | SOM | 0.4069 | 0.0045 |
| sp. richness Undefined saprotrophs | root biomass | 0.4085 | 0.0044 |
| sp. richness Undefined saprotrophs | soil moisture | -0.5894 | < 0.001 |
| sp. richness Undefined saprotrophs | total soil C | 0.4266 | 0.0028 |
| sp. richness Undefined saprotrophs | total soil N | 0.4035 | 0.0049 |
| sp. richness Undefined saprotrophs | soil pH | -0.6236 | < 0.001 |
| sp. richness Wood saprotrophs | soil moisture | -0.3557 | 0.0141 |
| sp. richness Wood saprotrophs | soil pH | -0.354 | 0.0132 |

**Table S6. Site characteristics**

A. Measured vegetation variables in the sampled plots (2x2m). The data is presented as average values for each of the three blocks of every grassland (tovetorp, amtvik) and elevation (high, low) (n=4). TH = Tovetorp High; TL = Tovetorp Low; AM = Amtvik High; AL = Amtvik Low. All data was collected in 2019.

| block | site | %dead plant material | %forbs | %grasses | %legumes | %mosses | %woody |
| --- | --- | --- | --- | --- | --- | --- | --- |
| 1 | TH | 27,9 | 9,1 | 47,7 | 0,7 | 14,6 | 0 |
| 2 | TH | 22,1 | 13,8 | 15,6 | 1,3 | 47,1 | 0 |
| 3 | TH | 30,9 | 3,4 | 14,0 | 5,2 | 46,5 | 0 |
| 4 | TL | 18,4 | 13,9 | 35,1 | 0,1 | 32,5 | 0 |
| 5 | TL | 44,2 | 0,3 | 54,1 | 0,2 | 1,1 | 0 |
| 6 | TL | 25,0 | 1,3 | 40,9 | 0,9 | 31,8 | 0 |
| 7 | AH | 24,2 | 23,0 | 48,2 | 3,7 | 0,8 | 0 |
| 8 | AH | 25,3 | 18,8 | 46,6 | 7,9 | 1,4 | 0 |
| 9 | AH | 28,8 | 16,3 | 48,8 | 5,1 | 1,0 | 0 |
| 10 | AL | 11,7 | 18,3 | 62,3 | 5,3 | 2,4 | 0 |
| 11 | AL | 10,7 | 20,7 | 27,6 | 12,7 | 28,3 | 0 |
| 12 | AL | 6,4 | 18,0 | 25,6 | 5,4 | 38,3 | 6,4 |

| block | site | distance to closest tree (m) | aboveground plant biomass (g/m^2^) | number of plant species | Shannon index plants |
| --- | --- | --- | --- | --- | --- |
| 1 | TH | 59 | 351 | 28 | 2,67 |
| 2 | TH | 70 | 383 | 31 | 2,81 |
| 3 | TH | 85 | 383 | 30 | 2,77 |
| 4 | TL | 45 | 401 | 22 | 2,27 |
| 5 | TL | 65 | 506 | 24 | 2,05 |
| 6 | TL | 60 | 486 | 20 | 1,69 |
| 7 | AH | 8,5 | 492 | 27 | 2,26 |
| 8 | AH | 7,5 | 486 | 28 | 2,55 |
| 9 | AH | 21 | 451 | 22 | 2,09 |
| 10 | AL | 6 | 299 | 39 | 2,58 |
| 11 | AL | 8 | 322 | 36 | 2,68 |
| 12 | AL | 10 | 368 | 37 | 2,69 |

B. Measured soil variables in the sampled plots (2x2m). The data is presented as average values for each of the three blocks of every grassland (tovetorp, amtvik) and elevation (high, low) (n=4). TH = Tovetorp High; TL = Tovetorp Low; AM = Amtvik High; AL = Amtvik Low. All data was collected in 2019.

| sample | block | depth | site | % soil organic matter (SOM) | volumetric soil moisture | soil C (µgC/mg soil) | soil N (µgN/mg soil) | root biomass (g/m^2^) | Soil pH |
| --- | --- | --- | --- | --- | --- | --- | --- | --- | --- |
| 1 | 1 | 0-10cm | TH | 1,96 | 7,36 | 21,43 | 1,86 | 821,76 | 5,87 |
| 2 | 1 | 10-20cm | TH | 1,78 | 13,94 | 13,74 | 1,28 | 46,45 | 5,95 |
| 3 | 1 | 20-30cm | TH | 1,51 | 23,41 | 12,96 | 1,26 | 9,05 | 6,19 |
| 4 | 1 | 40-50cm | TH | 1,18 | 34,49 | 4,80 | 0,60 | 0,00 | 6,99 |
| 5 | 2 | 0-10cm | TH | 3,07 | 7,14 | 20,15 | 1,78 | 576,88 | 5,88 |
| 6 | 2 | 10-20cm | TH | 2,02 | 10,82 | 18,40 | 1,67 | 41,87 | 6,02 |
| 7 | 2 | 20-30cm | TH | 1,72 | 18,01 | 12,75 | 1,25 | 22,08 | 6,14 |
| 8 | 2 | 40-50cm | TH | 1,77 | 31,25 | 10,78 | 1,10 | 0,00 | 6,82 |
| 9 | 3 | 0-10cm | TH | 1,66 | 10,45 | 20,89 | 1,76 | 710,76 | 5,92 |
| 10 | 3 | 10-20cm | TH | 1,55 | 14,02 | 13,43 | 1,24 | 14,72 | 6,00 |
| 11 | 3 | 20-30cm | TH | 1,69 | 20,68 | 9,15 | 0,91 | 24,67 | 6,37 |
| 12 | 3 | 40-50cm | TH | 1,02 | 28,62 | 3,91 | 0,39 | 0,00 | 6,61 |
| 13 | 4 | 0-10cm | TL | 5,50 | 6,52 | 39,34 | 3,36 | 1004,97 | 5,42 |
| 15 | 4 | 20-30cm | TL | 2,92 | 19,12 | 25,77 | 2,27 | 33,52 | 5,66 |
| 16 | 4 | 40-50cm | TL | 1,90 | 35,83 | 21,41 | 1,84 | 0,00 | 6,93 |
| 17 | 5 | 0-10cm | TL | 2,95 | 8,04 | 36,43 | 3,14 | 2040,18 | 5,65 |
| 18 | 5 | 10-20cm | TL | 3,18 | 13,14 | 34,01 | 2,97 | 219,22 | 5,59 |
| 19 | 5 | 20-30cm | TL | 2,66 | 21,38 | 24,46 | 2,15 | 136,96 | 5,69 |
| 20 | 5 | 40-50cm | TL | 2,43 | 30,64 | 22,84 | 2,00 | 0,00 | 6,05 |
| 21 | 6 | 0-10cm | TL | 2,93 | 8,86 | 31,09 | 2,71 | 2630,10 | 5,95 |
| 22 | 6 | 10-20cm | TL | 2,83 | 13,11 | 28,80 | 2,54 | 175,45 | 5,80 |
| 23 | 6 | 20-30cm | TL | 2,49 | 20,95 | 23,53 | 2,14 | 107,72 | 5,85 |
| 24 | 6 | 40-50cm | TL | 1,51 | 31,40 | 18,25 | 1,66 | 0,00 | 6,12 |
| 25 | 7 | 0-10cm | AH | 3,42 | 10,76 | 27,53 | 2,18 | 1122,84 | 5,50 |
| 26 | 7 | 10-20cm | AH | 1,92 | 14,33 | 25,64 | 2,01 | 65,55 | 5,56 |
| 27 | 7 | 20-30cm | AH | 1,50 | 21,62 | 12,11 | 1,03 | 2,88 | 6,29 |
| 28 | 7 | 40-50cm | AH | 1,07 | 32,29 | 2,58 | 0,25 | 0,00 | 6,75 |
| 29 | 8 | 0-10cm | AH | 2,15 | 6,89 | 30,61 | 2,28 | 552,72 | 5,66 |
| 30 | 8 | 10-20cm | AH | 1,90 | 9,35 | 19,83 | 1,70 | 16,81 | 5,71 |
| 31 | 8 | 20-30cm | AH | 1,32 | 16,40 | 12,84 | 1,10 | 0,40 | 6,27 |
| 32 | 8 | 40-50cm | AH | 1,15 | 29,01 | 7,29 | 0,63 | 0,00 | 6,63 |
| 33 | 9 | 0-10cm | AH | 4,42 | 9,59 | 24,78 | 2,07 | 1303,86 | 5,59 |
| 34 | 9 | 10-20cm | AH | 2,34 | 12,83 | 38,60 | 2,62 | 50,93 | 6,05 |
| 35 | 9 | 20-30cm | AH | 1,16 | 20,91 | 17,68 | 1,54 | 0,40 | 6,23 |
| 36 | 9 | 40-50cm | AH | 1,61 | 32,04 | 5,25 | 0,49 | 0,00 | 6,23 |
| 37 | 10 | 0-10cm | AL | 2,46 | 10,84 | 32,98 | 2,49 | 932,66 | 5,69 |
| 38 | 10 | 10-20cm | AL | 1,47 | 12,25 | 31,66 | 2,42 | 406,90 | 5,72 |
| 39 | 10 | 20-30cm | AL | 1,54 | 17,54 | 22,08 | 1,78 | 61,37 | 6,29 |
| 40 | 10 | 40-50cm | AL | 2,99 | 33,60 | 11,61 | 0,94 | 0,00 | 6,28 |
| 41 | 11 | 0-10cm | AL | 2,70 | 11,75 | 32,27 | 2,45 | 929,88 | 5,48 |
| 42 | 11 | 10-20cm | AL | 1,74 | 13,86 | 26,47 | 2,07 | 67,54 | 5,53 |
| 43 | 11 | 20-30cm | AL | 1,40 | 20,17 | 19,43 | 1,35 | 25,96 | 6,16 |
| 44 | 11 | 40-50cm | AL | 1,35 | 33,99 | 17,47 | 1,38 | 0,00 | 6,37 |
| 45 | 12 | 0-10cm | AL | 3,20 | 12,72 | 27,71 | 2,18 | 967,97 | 5,66 |
| 46 | 12 | 10-20cm | AL | 2,06 | 14,64 | 17,60 | 1,45 | 177,44 | 6,02 |
| 47 | 12 | 20-30cm | AL | 1,35 | 20,30 | 16,39 | 1,27 | 57,89 | 6,59 |
| 48 | 12 | 40-50cm | AL | 1,64 | 36,30 | 6,75 | 0,56 | 0,00 | 6,55 |

## Figures

### Experimental setup


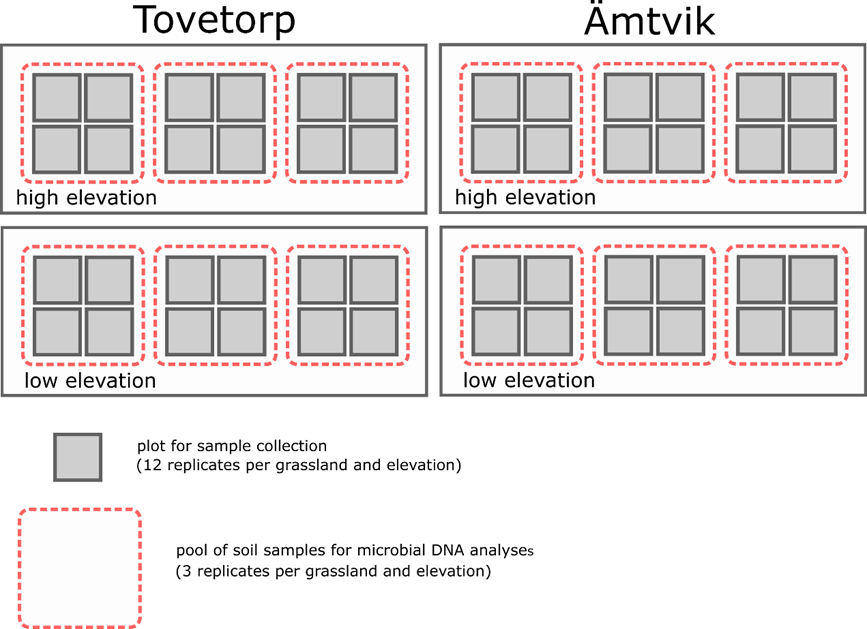


Figure S1. Diagram of the experimental setup

### Species accumulation plots
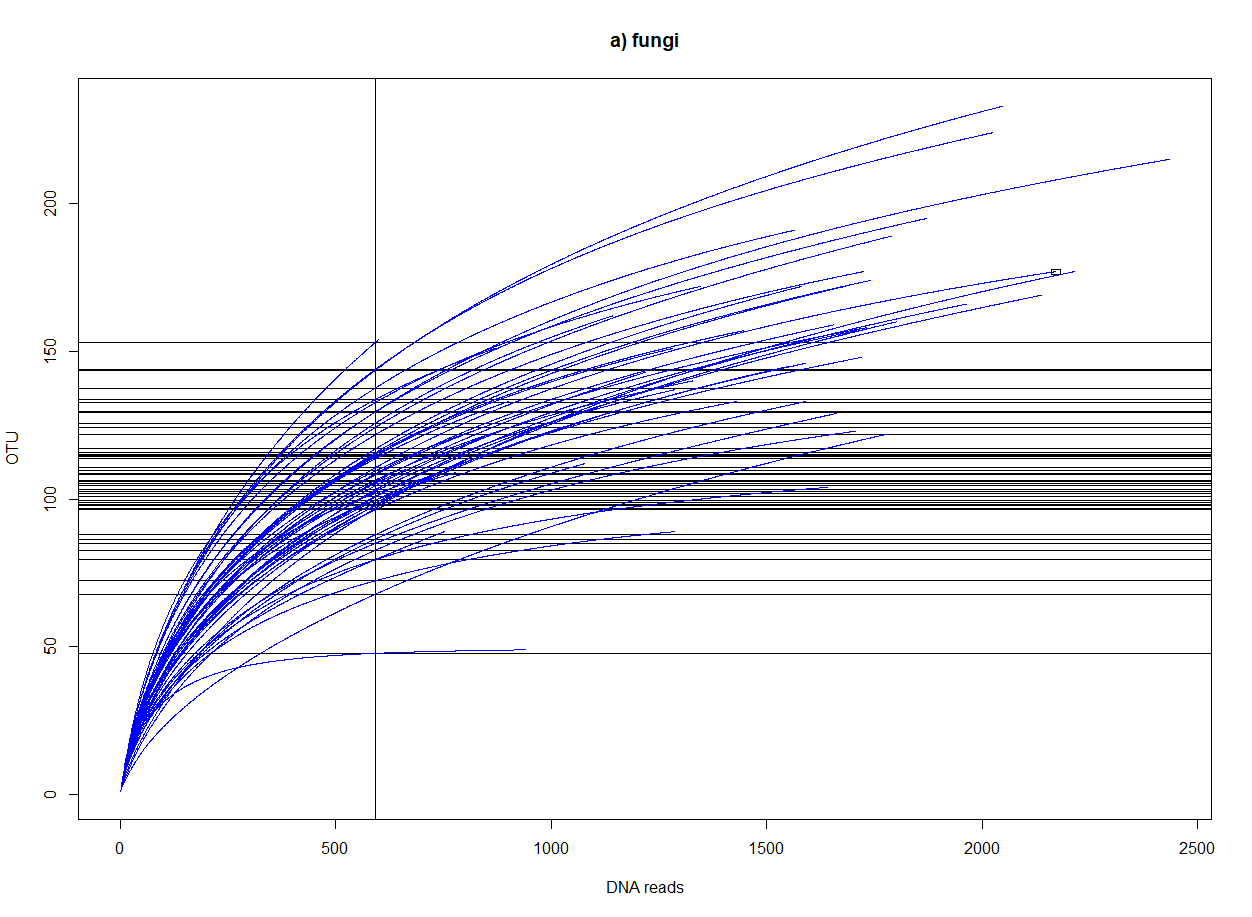

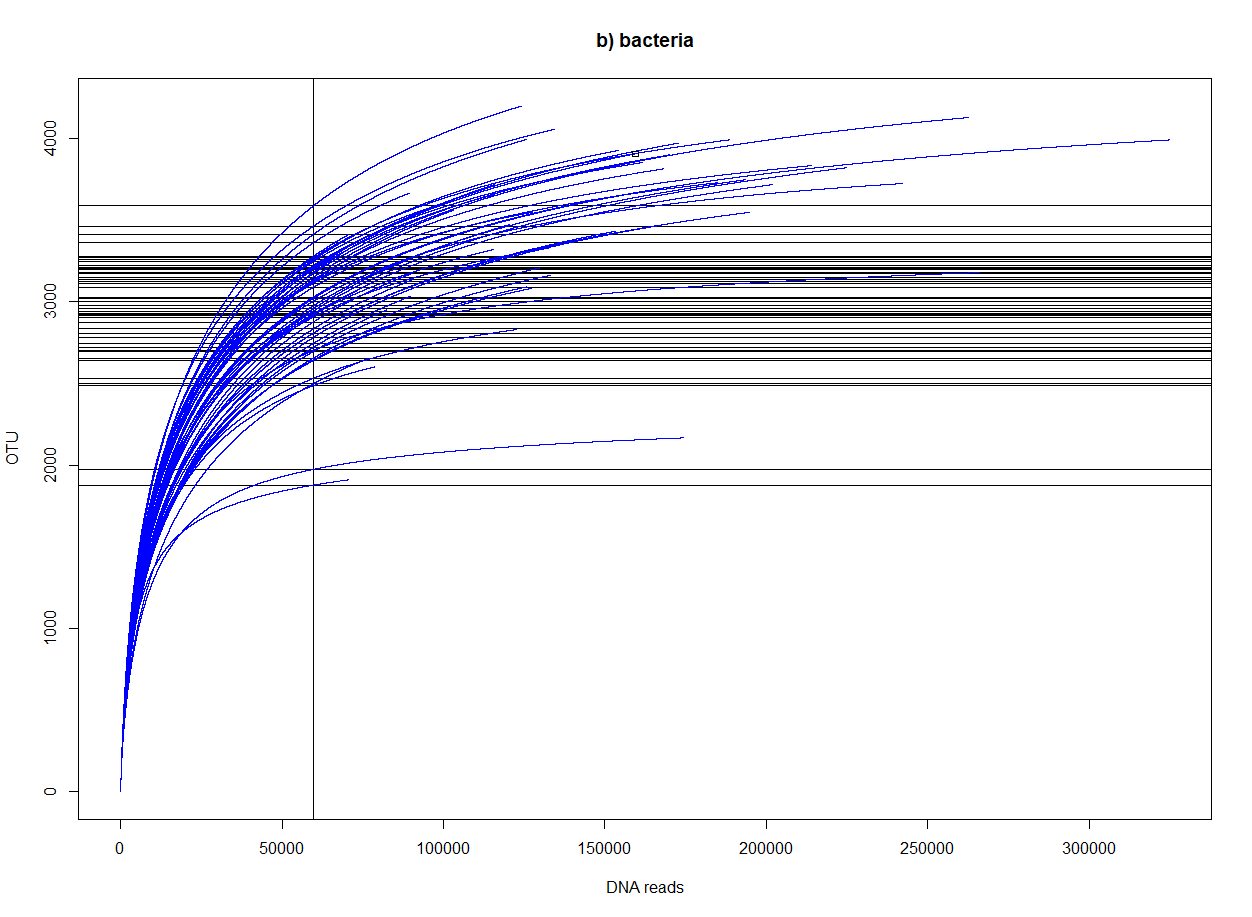


Figure S2. Species accumulation curves for (a) fungal OTUs and (b) bacterial OTUs for each sample.

### Ordination analyses

###
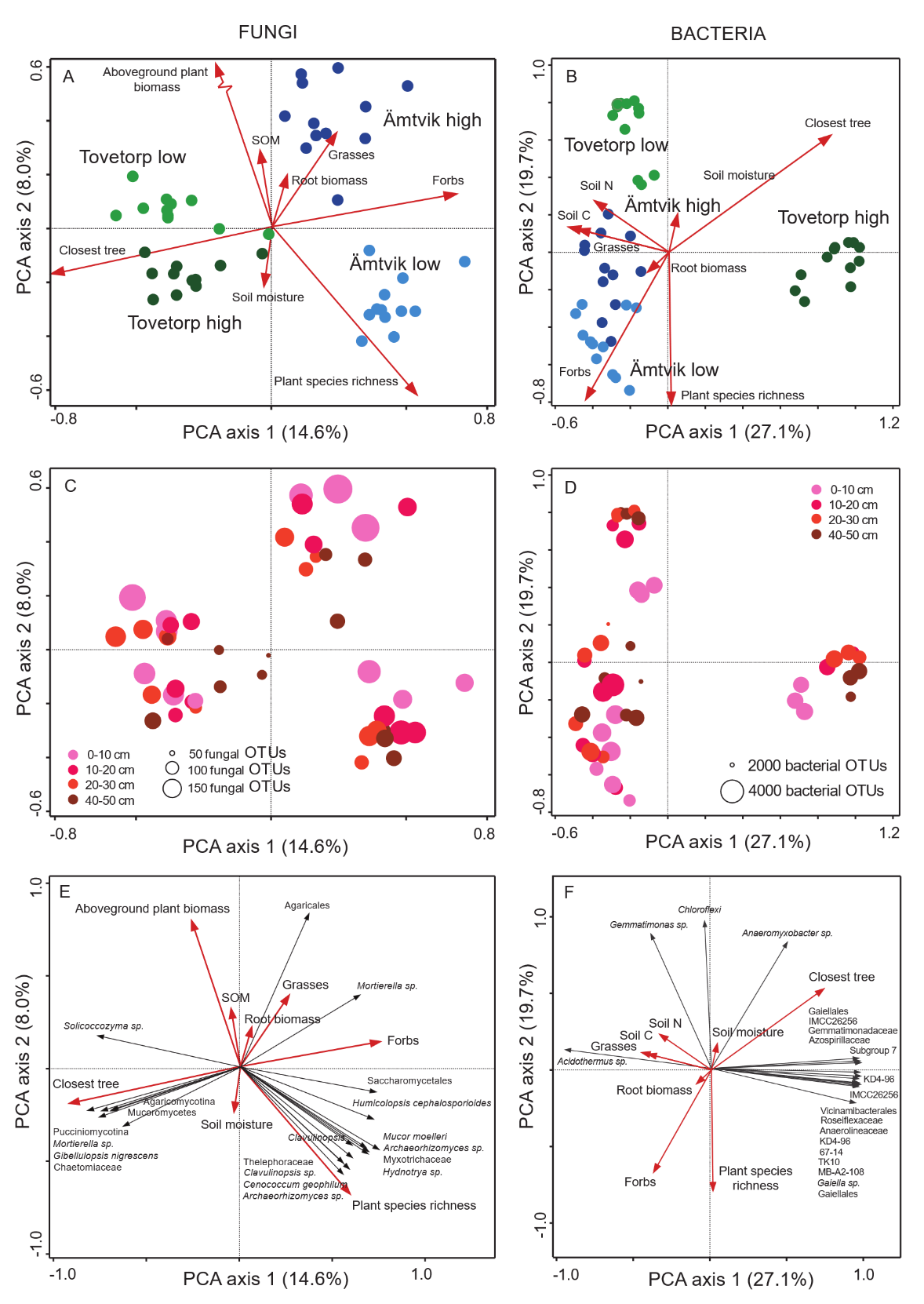
PCA

Figure S3. Variation in soil fungal (left panels) and bacterial (right panels) community composition in two grasslands (Tovetorp and Ämtvik) and catenary positions (high, low). Community composition across 47 (fungi) and 48 (bacteria) samples is visualized by (a-d) sample plots and (e, f) species plots of a PCA based either on PacBio sequencing of amplified ITS2 markers (fungi) or Illumina MiSeq sequencing of amplified 16S rRNA gene markers (bacteria). The PCAs were based on 780 identified fungal operation taxonomical units (OTUs), data not rarefied, and on 7115 identified bacterial OTUs, data rarefied although only the 30 most abundant OTUs are shown in the species plots. Circles are color coded according to: (a, b) grassland and elevation, and (c, d) soil sampling depth. The size of circles in (c, d) corresponds to the number of OTUs in each sample. In (a, e) red vectors indicate direction and degree of correlation between the PCA axes and aboveground plant biomass, plant root biomass, number of plant species, proportion of grasses and forbs, distance to closest tree, soil moisture, and SOM, added to the unconstrained analysis as supplementary variables. In (b, f) red vectors indicate direction and degree of correlation between the PCA axes and plant root biomass, number of plant species, proportion of forbs, distance to closest tree, soil moisture, and SOM.

### NMDS

Figure S4. Variation in (a) soil fungal and (b) bacterial community composition in two grasslands (Tovetorp and Ämtvik, Sweden) with two elevations (high and low). Community composition is visualized by samples plots of nonlinear multidimensional scaling analyses (NMDS) summarizing the similarities in species composition between samples based on PacBio sequencing of amplified ITS2 markers for fungi (a), and Illumina MySeq sequencing of amplified 16S rRNA gene for bacteria (b). The NMDS was based on 780 identified operational taxonomic units (OTUs), data not rarefied for fungi, and on 7115 identified OTUs, data rarefied, for bacteria. Circles are color coded according to: grassland and elevation. The first two axes explained 81.0 and 75.3 % of the total variation of 47.0 and 48.0, for fungi and bacteria respectively.

### PCA, corrected for qPCR data

**
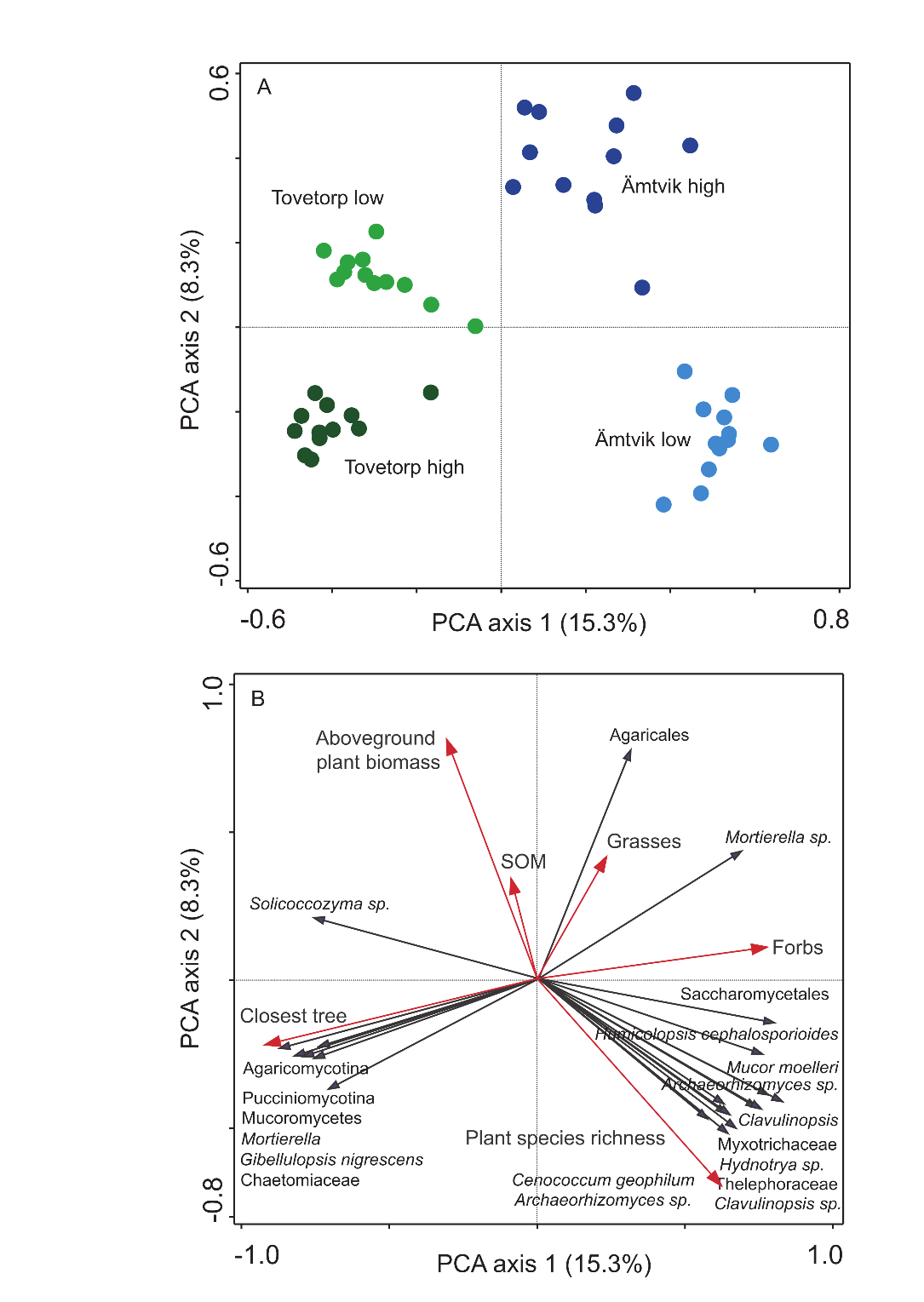
**

Figure S5. Variation in soil fungal community composition in two grasslands (Tovetorp and Ämtvik, Sweden) when accounting for total fungal biomass in each sample. Community composition across 48 samples is visualized by (a) a sample plot and (b) a species plot of a principal components analysis (PCA) based on PacBio sequencing of amplified ITS2 markers. The PCA was based on 780 identified fungal operational taxonomic units (OTU), and relative abundance data was recalculated based on the number of DNA copies per dry weight of soil obtained through qPCR. Circles are color coded according to: (a) grassland and elevation. In (b) red vectors indicate direction and degree of correlation between the PCA axes and aboveground plant biomass, number of plant species, proportion of grasses and forbs, distance to closest tree, and SOM. For the species plot only the 30 most abundant OTUs are shown. Axes 1 and 2 explained 15.3 and 8.3 %, respectively, of the total variation of 43963, when accounting for sequencing depth in each sample.

**
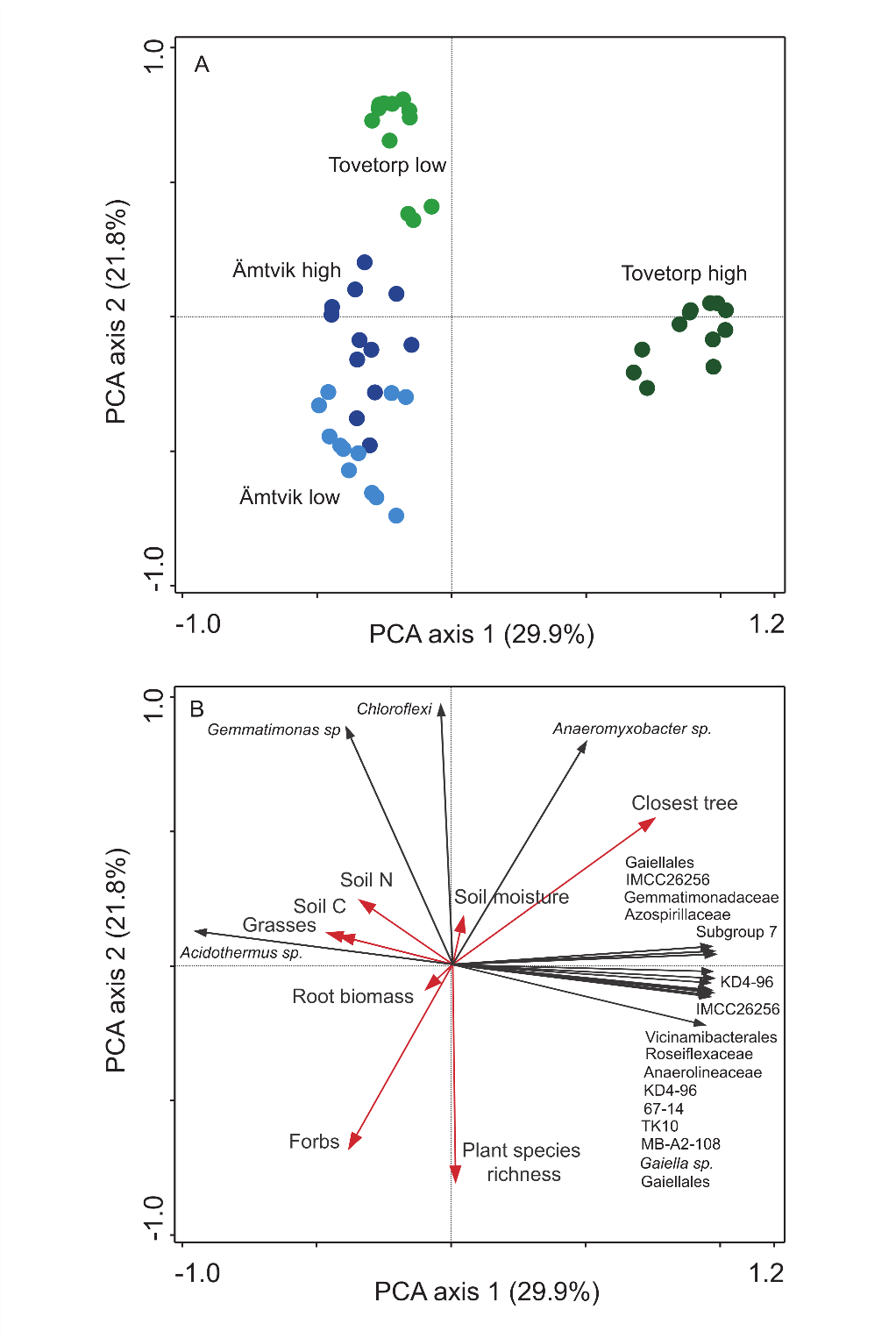
**

Figure S6. Variation in bacterial community composition in two grasslands (Tovetorp and Ämtvik, Sweden) when accounting for total bacterial biomass in each sample. Community composition across 48 samples is visualized by (a) a sample plot and (b) a species plot of a principle components analysis (PCA) based on Illumina MySeq sequencing of amplified 16S rRNA gene markers. The PCA was based on 7115 identified bacterial OTUs, and relative abundance data was recalculated based on quantification of bacterial 16Sin each sample. Circles are color coded according to: (a) grassland and elevation. In (b) red vectors indicate direction and degree of correlation between the PCA axes and root biomass, number of plant species, proportion of grasses and forbs, distance to closest tree, soil moisture, soil C and soil N. For the species plot only the 30 most abundant OTUs are shown. Axes 1 and 2 explained 29.9 and 21.8 %, respectively, of the total variation of 50748, when accounting for sequencing depth in each sample.

### RDA – depthwise analyses


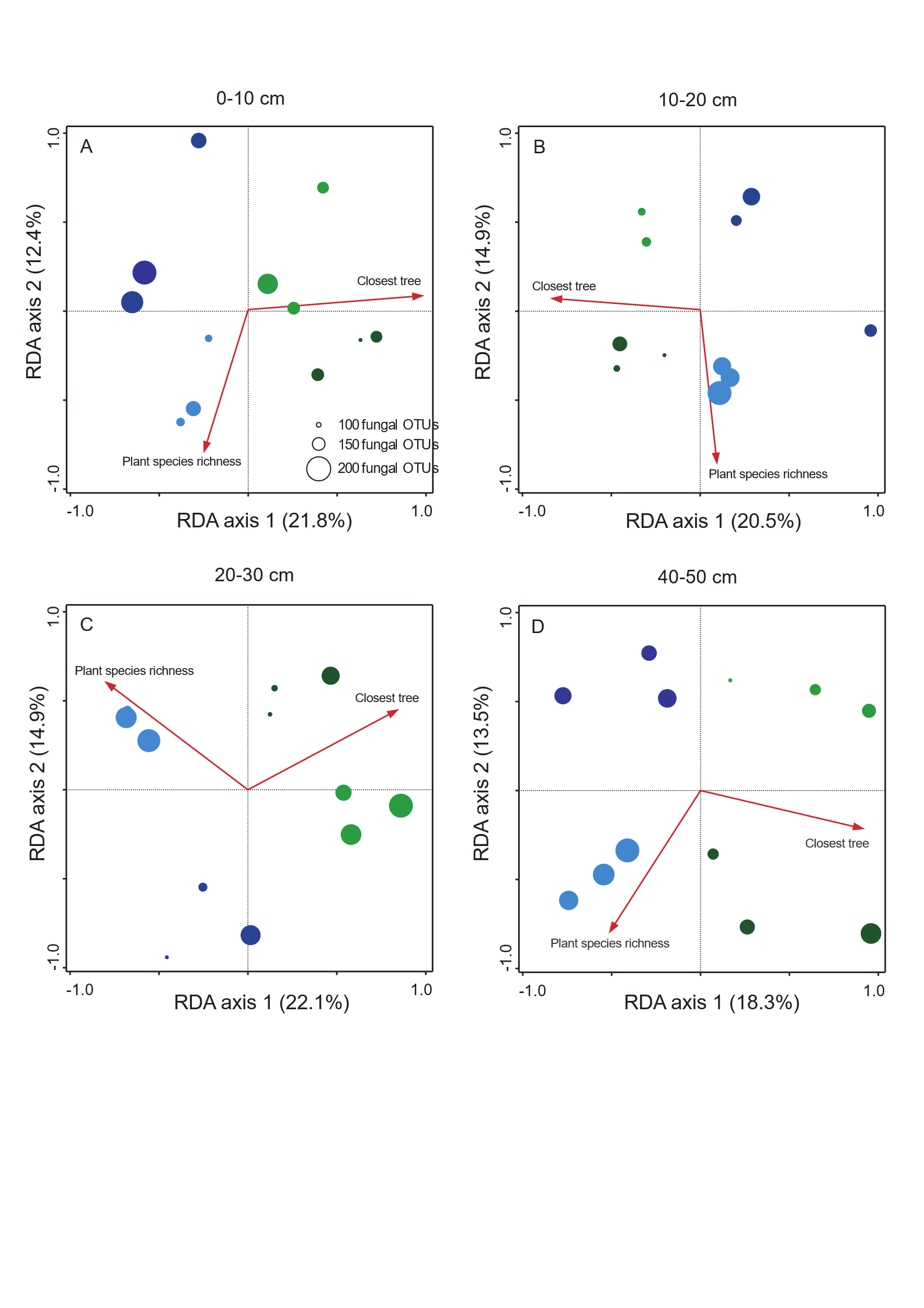


Figure S7**.** Variation in soil fungal community composition in two grasslands (Tovetorp and Ämtvik, Sweden) at four different soil depths visualized by sample plots of redundancy analyses (RDA) based on PacBio sequencing of amplified ITS2 markers. Soil depths corresponds to (a) 0-10 cm, (b) 10-20 cm, (c) 20-30 cm and (d) 40-50 cm. The RDAs included 780 identified fungal operational taxonomic units (OTU), data not rarefied. Circles are color coded according to grassland and elevation, and the size of circles corresponds to the number of fungal OTUs in each sample. Red vectors represent constraining variables and indicate direction and degree of correlation between RDA axes and number of plant species and the distance to the closest tree. Axes 1 and 2 explained 33.7, 35.4, 37.0 and 31.8% of the total variation for 0-10 cm, 10-20 cm, 20-30 cm and 40-50 cm, respectively, when accounting for sequencing depth in each sample. The total variation was 9518, 8332, 8035 and 8567 for respective soil depth.

Figure S8. Variation in soil bacterial community composition in two grasslands (Tovetorp and Ämtvik, Sweden) at four different soil depths visualized by sample plots of redundancy analyses (RDA) based on Illumina MySeq sequencing of amplified 16S markers. Soil depths corresponds to (a) 0-10 cm, (b) 10-20 cm, (c) 20-30 cm and (d) 40-50 cm. The RDAs included 7115 identified bacterial OTUs, data rarefied. Circles are color coded according to grassland and elevation, and the size of circles corresponds to the number of bacterial OTUs in each sample. Red vectors represent constraining variables and indicate direction and degree of correlation between RDA axes and root biomass, number of plant species and the distance to the closest tree. Axes 1 and 2 explained 57.2, 56.3, 59.0 and 57.0 % of the total variation for 0-10 cm, 10-20 cm, 20-30 cm and 40-50 cm, respectively, when accounting for sequencing depth in each sample. The total variation was 7597, 8180, 8758 and 10872 for respective soil depth.

## Mvabund analyses – community composition

Figure S9. Abundance of (a) fungal functional groups, (b) fungal divisions and (c) bacteria phyla, measured in DNA sequence counts, corrected by the amount of DNA obtained through qPCR.
